# Lid opening and conformational stability of T1 Lipase is mediated by increasing chain length polar solvents

**DOI:** 10.7717/peerj.3341

**Published:** 2017-05-18

**Authors:** Jonathan Maiangwa, Mohd Shukuri Mohamad Ali, Abu Bakar Salleh, Raja Noor Zaliha Raja Abd Rahman, Yahaya M. Normi, Fairolniza Mohd Shariff, Thean Chor Leow

**Affiliations:** 1 Department of Cell and Molecular Biology/Enzyme Microbial Technology Research center/Faculty of Biotechnology and Biomolecular Science, Universiti Putra Malaysia, Serdang, Serlangor, Malaysia; 2 Department of Biochemistry/Enzyme Microbial Technology Research center/Faculty of Biotechnology and Biomolecular Science, Universiti Putra Malaysia, Serdang, Selangor, Malaysia; 3 Department of Microbiology/Enzyme Microbial Technology Research center/Faculty of Biotechnology and Biomolecular Science, Universiti Putra Malaysia, Serdang, Selangor, Malaysia; 4 Department of Cell and Molecular Biology/Enzyme and Microbial Technology Research center/Faculty of Biotechnology and Biomolecular Science/Institute of Bioscience, Universiti Putra Malaysia, Serdang, Selangor, Malaysia

**Keywords:** Molecular dynamics, Organic solvent mixtures, Structure conformation, Interactions, Residue fluctuations, Cross correlations

## Abstract

The dynamics and conformational landscape of proteins in organic solvents are events of potential interest in nonaqueous process catalysis. Conformational changes, folding transitions, and stability often correspond to structural rearrangements that alter contacts between solvent molecules and amino acid residues. However, in nonaqueous enzymology, organic solvents limit stability and further application of proteins. In the present study, molecular dynamics (MD) of a thermostable *Geobacillus zalihae* T1 lipase was performed in different chain length polar organic solvents (methanol, ethanol, propanol, butanol, and pentanol) and water mixture systems to a concentration of 50%. On the basis of the MD results, the structural deviations of the backbone atoms elucidated the dynamic effects of water/organic solvent mixtures on the equilibrium state of the protein simulations in decreasing solvent polarity. The results show that the solvent mixture gives rise to deviations in enzyme structure from the native one simulated in water. The drop in the flexibility in H_2_O, MtOH, EtOH and PrOH simulation mixtures shows that greater motions of residues were influenced in BtOH and PtOH simulation mixtures. Comparing the root mean square fluctuations value with the accessible solvent area (SASA) for every residue showed an almost correspondingly high SASA value of residues to high flexibility and low SASA value to low flexibility. The study further revealed that the organic solvents influenced the formation of more hydrogen bonds in MtOH, EtOH and PrOH and thus, it is assumed that increased intraprotein hydrogen bonding is ultimately correlated to the stability of the protein. However, the solvent accessibility analysis showed that in all solvent systems, hydrophobic residues were exposed and polar residues tended to be buried away from the solvent. Distance variation of the tetrahedral intermediate packing of the active pocket was not conserved in organic solvent systems, which could lead to weaknesses in the catalytic H-bond network and most likely a drop in catalytic activity. The conformational variation of the lid domain caused by the solvent molecules influenced its gradual opening. Formation of additional hydrogen bonds and hydrophobic interactions indicates that the contribution of the cooperative network of interactions could retain the stability of the protein in some solvent systems. Time-correlated atomic motions were used to characterize the correlations between the motions of the atoms from atomic coordinates. The resulting cross-correlation map revealed that the organic solvent mixtures performed functional, concerted, correlated motions in regions of residues of the lid domain to other residues. These observations suggest that varying lengths of polar organic solvents play a significant role in introducing dynamic conformational diversity in proteins in a decreasing order of polarity.

## Introduction

The most useful property of natively folded proteins is their evolved functions in both aqueous and nonaqueous media. The functional relevance of proteins is well established in aqueous media with water promoting folding, conformational mobility, and stability (Mattos & Ringe, 2001). Protein stability in nonaqueous organic solvents arises from a combination of factors, which are often difficult to experimentally probe. The intrinsic conformational properties and factors promoting the denaturation of proteins provide useful knowledge of the molecular events that occur when performing catalysis under non- and/or physiologic conditions (Papaleo et al., 2011; Trodler, Schmid & Pleiss, 2009; Wedberg, Abildskov & Peters, 2012; Kony, HuNenberger & Van Gunsteren, 2007; Soares, Teixeira & Baptista, 2003). Although most enzymes are denatured in organic solvent media, some enzymes retain stability in some organic solvents with seemingly improved versatility, catalysis, substrate selectivity and biotechnological applications (Carrea & Riva, 2000; Klibanov, 2001).

In principle, the molecular phenomena of protein stability in organic solvents have been investigated by recent advances in molecular dynamics (MD) to complement and guide experimental studies. As a result, the earliest studies provided useful information on dynamics and conformational landscapes of proteins in a wide variety of solvent types at the femtosecond to microsecond time scale. This makes it possible to extract detailed information on functionally important conformational behaviour that can influence biological functions of proteins (Burney & Pfaendtner, 2013; Oelmeier, Dismer & Hubbuch, 2012; Li et al., 2010; Soares, Teixeira & Baptista, 2003).

Due to the prime role played by proteins in biotechnological processes, the impact of organic solvents in substrate selectivity, enzyme solubility, and stability is crucial (Juhl et al., 2010; Kawata & Ogino, 2010). In this context, hydrolytic enzymes such as lipases have been considered to have both a sufficient conformational flexibility and stabilityto catalyse hydrolysis in organic media. Their broad range of properties and substrate specificities makes them prominent in most enzymatic reactions in nonaqueous reaction systems (Kapoor & Gupta, 2012). However, the stability of lipase particularly in hydrophilic organic solvents is reduced by the organic solvent penetration effect and greater tendency to strip tightly bound water from the enzyme molecules (Gorman & Dordick, 1992; Yang, Dordick & Garde, 2004). Also, since most lipases are considered to be medium-dependent, far too often organic solvents are involved in disrupting their structural interactions through a weakening of the hydrophobic effects and hydrogen-bonding interactions (Ji et al., 2010; Kony, HuNenberger & Van Gunsteren, 2007). Similarly, distortion of protein flexibility, displacement of the lid domain helices and unmasking the active site in organic solvents could be infrequently associated with the polarity of the solvent (Graber et al., 2003).

Different hydration studies and solvent dependent properties of enzymes in various solvents have been investigated by MD simulations (Micaelo et al., 2005; Micaelo & Soares, 2007). *Candida antartica* lipase B has been described in various solvents to show similar enzyme hydration with profound effects on the structural properties. Wedberg and co-workers (2012) suggest the overall flexibility increased with increasing solvent polarity and the organic solvents modulated the protein-bound water molecules. Lousa, Baptista & Soares (2012) carried out simulations on homologous proteases in water/ethanol solvent mixtures in which large conformational changes and penetration of organic solvent into the protein core destroyed the structure. Disruption of salt bridges as a result of the organic solvent effect is also a critical molecular determinant of stability. The potential use of nonaqueous structure-perturbing solvents like DMSO can also result in a cooperative transition of the protein to a new partially folded state (Bhattacharjya & Balaram, 1997).

**Figure 1:**
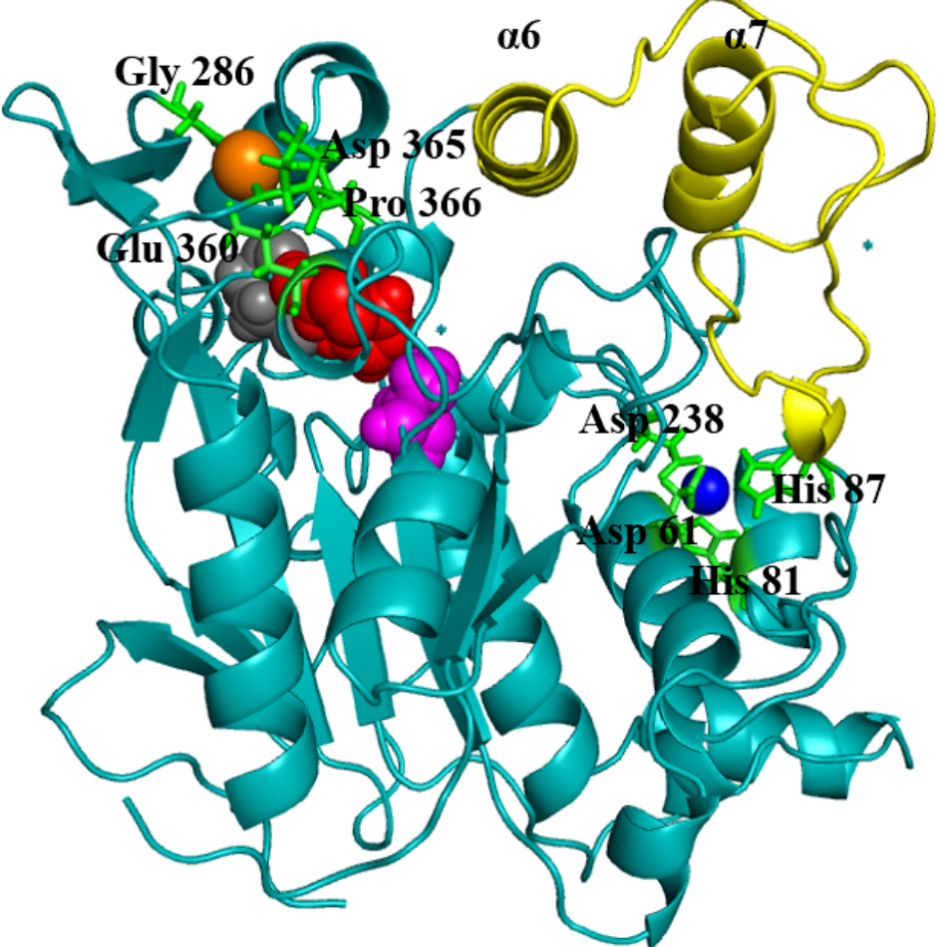
Structure of the closed conformation of T1. View of the T1 structure with different colored domains: (α/ β) hydrolase, *cyan*; cap domain helix α6 and α7 connected by the loop residues 175–230, *yellow*; the active site Ser113, *magenta*; Asp317, *red* and His358, *gray* spheres, respectively. Metal ions Zn^2+^, *blue*; Ca^2+^ *orange* are in spheres held together by a network of amino acid residues, *green*.

*Geobacillus zalihae* T1 lipase (a thermoalkaliphic lipase) is a 43 kDa protein that has been shown to catalyse the hydrolysis of long-chain triglycerides into fatty acids at a high temperature of ∼70°C (Leow et al., 2007). Industrially, this property makes it a potentially efficient biocatalyst for high temperature organic solvent media reactions. Structural resolution of T1 lipase has been determined at 1.5 Å in a closed conformation (Matsumura et al., 2008). The lipase shares the common canonical alpha/beta hydrolase fold (Ollis et al., 1992; Nardini & Dijkstra, 1999; Arpigny & Jaeger, 1999). The catalytic triad of Ser113, His358 and Asp317 are conserved in an active site shielded by a lid domain helix α6 and α7  (Fig. 1).

The metal ions (Ca^2+^ and Zn^2+^) present in T1 lipase are important local structural stabilizing elements of most native lipase structural folds (Gupta, Gupta & Rathi, 2004; Noble et al., 1993). These and other metal ions present in over 30% of known proteins function in catalysis and electron transfer and may play a key role in modulating protein folding and electrostatic stability (Andreini et al., 2008). The localization and coordination of the Ca^2+^ and Zn^2+^ share structural similarity with that of *Bacillus stearothermophilus* L1 lipase (Jeong et al., 2002). In this respect, the loop containing the active-site histidine (His358) is stabilized by an octahedral coordinated Ca^2+^, held by tight interactions with Asp365, Glu360, Pro366, and Gly286. The Zn^2+^ centre is held through Zn^2+^-coordinated tight interactions with Asp61, Asp238, His81, and His87. Temperature-induced MDsimulations of T1 lipase and activity regulation is Zn^2+^ dependent (Abdul Rahman et al., 2012). The mechanism of this Zn^2+^ effect has long been proposed to create structural modifications in lipase rather than affecting the catalytic active centre (Van Oort et al., 1989).

The phenomenal interfacial activation of T1 lipase in aqueous environments involves the movement of the helix α6 of the lid (Wang, Wei & Wang, 2010). Concurring with this similar characteristic, the helix α6 of *Bacillus stearothermophilus* L1 lipase, which is structurally similar to T1 lipase, was postulated to function as the lid in a closed conformation. However, in the open conformation, enzyme activation involves both dramatic conformational rearrangements of two lids (helix α6 and α7) covering the active site (Carrasco-López et al., 2009). The insights into T1 lipase activation events have not provided sufficient detail to suggest the overall structural property/dynamics that T1 lipase may adopt in a nonaqueous organic solvent environment.

This current study describes MD simulations of T1 lipase in water and five hydrophilic organic solvents/water mixtures. The solvents were investigated in their corresponding carbon chain lengths. Methanol and ethanol are quoted as the preferred solvents in the synthesis of carboxylic esters with lipase as biocatalysts. By also considering the thermostability of T1 lipase and the emerging use of these solvents in various bioconversion processes, we hope the scope of the current study will capture the physical features corresponding to experimental conditions. Details of insights into the general structural properties/structure dynamics, hydrophobicity, non-covalent interactions, solvent properties and lid activation are also investigated.

## Materials and Methods

### Preparation of protein and organic solvents structures

Crystal structure coordinates of the closed conformation of wild-type *Geobacillus zalihae* T1 lipase (PDB ID: 2DSN) (Matsumura et al., 2008) were obtained from the Protein Data Bank with a resolution of 1.5 Å. The starting coordinates, crystal water molecules and metal ions (Ca^2+^ and Zn^2+^) were reserved. The structures of organic aliphatic chain length solvents were taken from the PubChem Compound Database NCBI server (entries CID_6276, CID_263, CID_702, CID_887, CID_962, CID_1031), and are summarized in Table 1. To minimize the number of energetically unfavourable structural features, the protein structure was energy minimized and the hydrogen bonding network was optimized using YASARA force field (Krieger et al., 2004). The organic solvents were modelled using the automatic parameterization algorithm (AutoSMILES), which can derive force field parameters for organic molecules fully automatically (Wang et al., 2004; Jakalian, Jack & Bayly, 2002). This parametrization also considers force field parameters for small organic molecules.

**Table 1:** Properties of compounds used for MD simulation in this study.

Name of compound	ID	Abbreviations	No. of atoms	No. of Molecules used	Experimental density (g/cm^3^)	Simulated density (bar)
Water	CID_962	H_2_O	3	4,829	0.997	1
Methanol	CID_887	MtOH	6	2,144	0.7872	1
Ethanol	CID_702	EtOH	9	1,491	0.7873	1
Propanol	CID_1031	PrOH	12	1,165	0.8020	1
Butanol	CID_263	BtOH	15	949	0.8061	1
Pentanol	CID_6276	PtOH	18	806	0.8110	1

### Organic solvent stability assay of native T1 lipase

Organic solvent stability of T1 lipase activity was conducted by measuring the residual lipase activity quantitatively from the free fatty acid released based on the oleic acid standard according to Kwon & Rhee (1986). T1 lipase was purified by affinity chromatography with Ni^2+^ Sepharose loaded onto an XK16 column mounted on an AKTA Prime (GE healthcare). The purified enzyme (0.5 mg/mL) was incubated in five different polar solvents (methanol, ethanol, propanol, butanol, and pentanol) in a volume ratio of enzyme to organic solvent representing 25% and 50% final concentrations. The solution was thoroughly mixed and incubated at 70°C in a water bath shaker at 200 rpm for 1 h. Substrate emulsion (2.5 mL olive oil/Glycine-NaOH buffer pH 9 at 1:1 ratio, 20 μ*L* 20 mM CaCl_2_) was then added and incubated at 70°C with continuous shaking at 200 rpm for 30 min. The enzyme reaction in the emulsion system was stopped by adding 6 M HCl (1 mL) and isooctane (5 mL), and vortexed for 30 min. The upper isooctane layer containing the fatty acid was transferred to a test tube for analysis. Copper reagent (1 mL) (5% (w/v) copper (II) acetate-1-hydrate adjusted to pH 6.1 with pyridine) was added and vortexed for 30 s and allowed to stand for 2 h. The absorbance of the upper layer was read at 715 nm. One unit of lipase activity was defined as the releasing of 1 μmol of free fatty acid per minute.

### Preparation of the molecular dynamics setup

The YASARA Structure software package version 12.10.3 (Krieger et al., 2004), installed on a Dell XPS PC (Intel Core TM i7-6700 3.41 GHz), was used to perform all simulations. A rectangular cubic simulation box was put around the protein with a distance of 5.0 Å  around all atoms. Boundary conditions were set to periodic in the *x*, *y* and *z* directions. Due to the size and amount of solvent mixture molecules, the dimensions of the simulation box were determined automatically using the Auto parameter. This was done to avoid the risk of violating the minimum image convention due to interactions of the solvent molecules as they move across the periodic boundary. To make sure that the cell is neutral and protonation states of amino acid side-chains are assigned, a cell neutralization and pKa prediction experiment were performed using the AMBER03 force field (Duan et al., 2003). This places the ions at the locations of the lowest/highest electrostatic potential until the cell is neutralized and the requested ion mass fraction (0.9% NaCl) is reached. The water molecules were removed leaving the counterions. Each simulation box was then filled with the calculated copies of water/organic solvent molecules in order to achieve a concentration of 50% (Table 1). Since the number of solvent molecules required to attain a given activity cannot be established *ab-initio* (Wedberg, Abildskov & Peters, 2012), preliminary experimental assays guided the selection of the number of solvent molecules added in the system. The number of solvent molecules was adjusted by considering the experimental density at 298 K and 1 bar. For simulations involving pure water as a control, TIP3P water with flexible bonds modelled with AMBER03 force field was used (Miyamoto & Kollman, 1992) and the pressure rescaled until the experimental density of 0.997 g/ml was achieved.

### Molecular dynamic simulations protocol

The atoms of the protein structure in the different solvent mixtures in the simulation box were constrained (fixed). This is to equilibrate the solvent mixtures in the box and allow the relaxation of the solvent molecules and prevent large distortions to the protein. To remove bumps, delete unwanted water molecules and correct the covalent geometry, the atoms were unconstrained and the system setup was energy-minimized with the AMBER03 force field (Duan et al., 2003). The geometric parameters (dihedral angles, bond angles, and length) including Van der Waals and Coulomb interactions of the force field were truncated at a 7.86 Å  force cut-off. The Particle Mesh Ewald algorithm (Essman et al., 1995) was used to treat long-range electrostatic interactions with a grid spacing of <1 Å. After removal of conformational stress by a short steep descent minimization of 200 steps, the procedure continued by simulated annealing (time step 2 fs, atom velocities scaled down by 0.9 every 10th step) until convergence was reached. This final system setup was used for the production runs.

All MD simulations were carried out in three independent runs for each system over a 40 ns timescale using the AMBER03 force field on YASARA Structure software package. Different RandomSeed numbers based on the temperature control (343K) of YASARA documentation were assigned using a Maxwell–Boltzmann distribution to achieve different initial velocities for each replicate simulation. The velocities of the atoms were rescaled according to the Berendsen barostat as described by Krieger et al. (2004), and pH 9 was used in all solvents. The manometer pressure mode was used to control the pressure from the kinetic energy/virial and slowly rescale the simulation cell along the *X*, *Y*,  and *Z*-plane to smoothly approach 1 bar. Simulation snapshot coordinates were saved every 30 ps.

### Analysis of data and evaluation

Unless otherwise specified, all analyses of trajectories were performed from replicates of three different simulations run at different initial velocities. Molecular graphics were created with YASARA (http://www.yasara.org) and POVRay (http://www.povray.org) (Krieger & Vriend, 2014). The structural deviations of the backbone atoms (RMSD) of the simulation runs from the initial crystallographic structure, the B-factors from the root mean square fluctuations (RMSF), accessible solvent area (SASA) and secondary structure interactions were analysed by executing the YASARA macros protocol described in detail by Krieger & Vriend (2015). The RMSD is expressed by the difference between the Cartesian atom coordinates based on the selection formula

\begin{eqnarray*}RMSD=\sqrt{ \frac{\sum _{\mathfrak{i}=1}^{\mathfrak{n}}{\mathfrak{R} }_{\mathfrak{i}\ast }{\mathfrak{R} }_{\mathfrak{i}}}{\mathfrak{n}} } \end{eqnarray*}

where ‘*R*’ is the vector linking the ‘*n*’ corresponding atom pairs in space.

The B-factor as a measure of the RMSF was obtained with the following conversion factor

\begin{eqnarray*}& & RMS{F}_{\mathfrak{i}}=\sqrt{\sum _{j=1}^{3} \left( \frac{1}{N} \sum _{k=1}^{N}{P}_{ikj}^{2}-{P}_{ij}^{-2} \right) } \end{eqnarray*}

\begin{eqnarray*}& & B facto{r}_{i}= \frac{8}{3} { \left( \pi \ast RMS{F}_{i} \right) }^{2} \end{eqnarray*}

Where the RMSF of atom **i** in **Å**  and ***j*** runs over the three Cartesian components *x*, *y* and *z* of the atom position vector **P**, and **K** runs over the **N** coordinate sets. The solvent accessible area (SASA) was calculated using the default YASARA’s ‘Numeric’ algorithm with the radius of the water probe (solvation radii) set at 1.4 Å.

### Structural and conformational stability analysis

The contributions of hydrophobic and hydrogen bonds to protein stability was analysed. The hydrophobic interaction was calculated using YASARA’s knowledge-based potential. Hydrophobic atoms were identified and assigned an atom type. Type 1 carbons had ≥ 3 hydrogens (−CH_3_), type 2 had two hydrogens or one hydrogen plus three carbons bound (−CH_2_ −, HCC3) and type 3 had aromatic rings with only carbons and hydrogens bound. The interaction range was determined based on the minimum distance (where the energy becomes positive due to clashes), optimum distance (the energy minimum) and maximum distance (where energies become positive due to the cost of creating a vacuum between the atoms). Hydrophobicity based on the Eisenberg hydrophobicity scale (Eisenberg, Weiss & Terwilliger, 1984) was mapped on the surface using PyMOL 1.7.2.1 (DeLano, 2002).

The hydrogen bonds were analysed based on YASARA’s definition of hydrogen bond formation, whereby the analysis of bond energy as a function of the H-bond-acceptor distance in Å  and two scaling factors is derived by the formula:

\begin{eqnarray*}EnergyHBo=25\ast \frac{2.6-max \left( {Dis}_{H-{A}^{2.1}} \right) }{0.5} \ast {Scale}_{D-H-A}\ast {Scale}_{H-A-X}. \end{eqnarray*}

The secondary structure analysis was carried out using the Kabsch & Sander (1983) algorithm incorporated in their Dictionary of Secondary Structure for Proteins (DSSP) program to analyse the variation of protein secondary structure changes. The secondary structure assignment algorithm program installed onto YASARA was used to analyse the change in secondary structure contents.

### Time-correlated atomic motions

To identify correlated and concerted motions, we calculated the dynamic cross-correlation map (DCCM), which is a matrix representation of the time-correlated information between protein atoms ***i*** and ***j***, (*c*_*ij*_) (Hünenberger, Mark & Van Gunsteren, 1995). Dynamic cross-correlation maps were used to detect time-correlated motions in the protein as implemented by Krieger et al. (2004) in YASARA. Dynamic cross-correlation matrices between units ***i*** and ***j*** were obtained with the following expression:

\begin{eqnarray*}DCC{M}_{i,j}= \frac{ \left\langle {\vec{d}}_{i}.{\vec{d}}_{j} \right\rangle }{\sqrt{ \left\langle {d}_{i}^{2} \right\rangle } \left\langle {d}_{j}^{2} \right\rangle } \end{eqnarray*}

Where ***d*** is the displacement between the current position and the average position of all selected pairs of atoms and how their movements correlate. The angle brackets indicate the average over all trajectories. The values in the DCCM range from −1 (perfectly anti-correlated) to +1 (perfectly correlated). The values along the diagonal are always +1 (because the motion is perfectly correlated to itself).

## Results and Discussion

### Stability property of MD simulations

**Figure 2:**
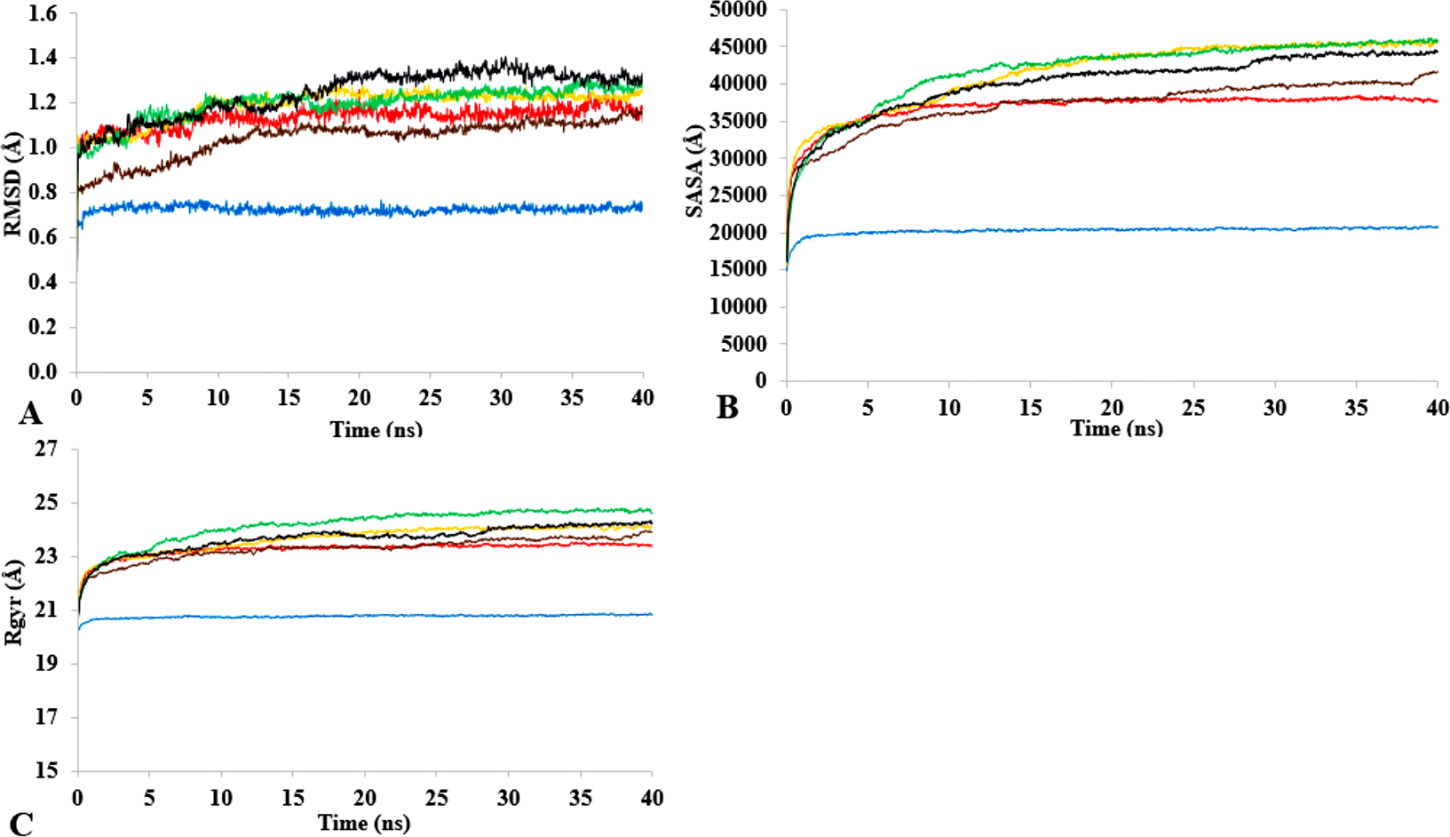
The time dependence of the mean average of replicates root mean square deviations (rmsd) of backbone atoms for the 40 ns simulations of T1 lipase (A). The Solvent accessible surface area (B) showed higher hydrophobic area of residues according to solvent polarity. The Radius of gyration (C) shows the same level of compactness among solvents compared to H_2_O. Both properties are calculated from mean average of three replicates of 40 ns simulations of solvent mixtures H_2_O (blue), MtOH-H_2_O (red), EtOH-H_2_O (brown), PrOH-H_2_O (yellow), BtOH-H_2_O (green), PtOH-H_2_O (black).

T1 lipase (Fig. 1) is considered a model lipase that could meet the demands of industrial biocatalysts. The general essential dynamics in identifying the structural changes that occurred during the molecular dynamics simulations over the timescale of 40 ns describe the important contributions of polar organic solvents to the general stability of T1 lipase. The time-average variations of backbone RMSD from the initial protein structure of T1 lipase simulations in water-organic solvent environments over a timescale of 40 ns are reported in Fig. 2 and Fig. S1. For comparisons, the simulations in water are also reported. The overall RMSDs are found to have a displacement difference across all solvent mixtures as they approach a point of convergence at 40 ns for the most equilibrated parts. Variations in RMSD for the simulations in H_2_O maintain a stable state and appear well equilibrated. The higher average RMSD values for simulations in solvent mixtures compared to water in Table 2 indicated structural differences in the protein backbone in these media. Accordingly, the RMSD of the backbone atoms increases with decreasing polarity of solvent mixtures. The conformational differences in RMSD are consistent with the role of solvents in having a different degree of hydration and dehydration effects on enzymes because the lower polarity of longer carbon chain length solvent mixtures can weaken interactions stabilizing the structure (Diaz-Garcia & Valencia-Gonzalez, 1995). The SASA (Fig. 2B and Fig. S2) deviates among all organic solvent mixture simulations. Higher SASA in some solvents can result in increased hydrophobic areas of residues, ultimately reducing the compactness of the hydrophobic core of the protein. Gyration radius (Rgyr) (Fig. 2C and Fig. S3) reveals the compactness of the simulations, and the increase in the Rgyr of all solvent mixtures is consistent with the solvation of charged and polar residues on the protein surface (Arcangeli, Bizzarri & Cannistraro, 2001). The SASA and Rgyr reported here are consistent with previous findings where the stable curves at equilibration keeps the T1 lipase in a suitable configuration that will favour its catalytic activity (Wang, Wei & Wang, 2010).

### Flexibility of T1 in solvents

**Figure 3:**
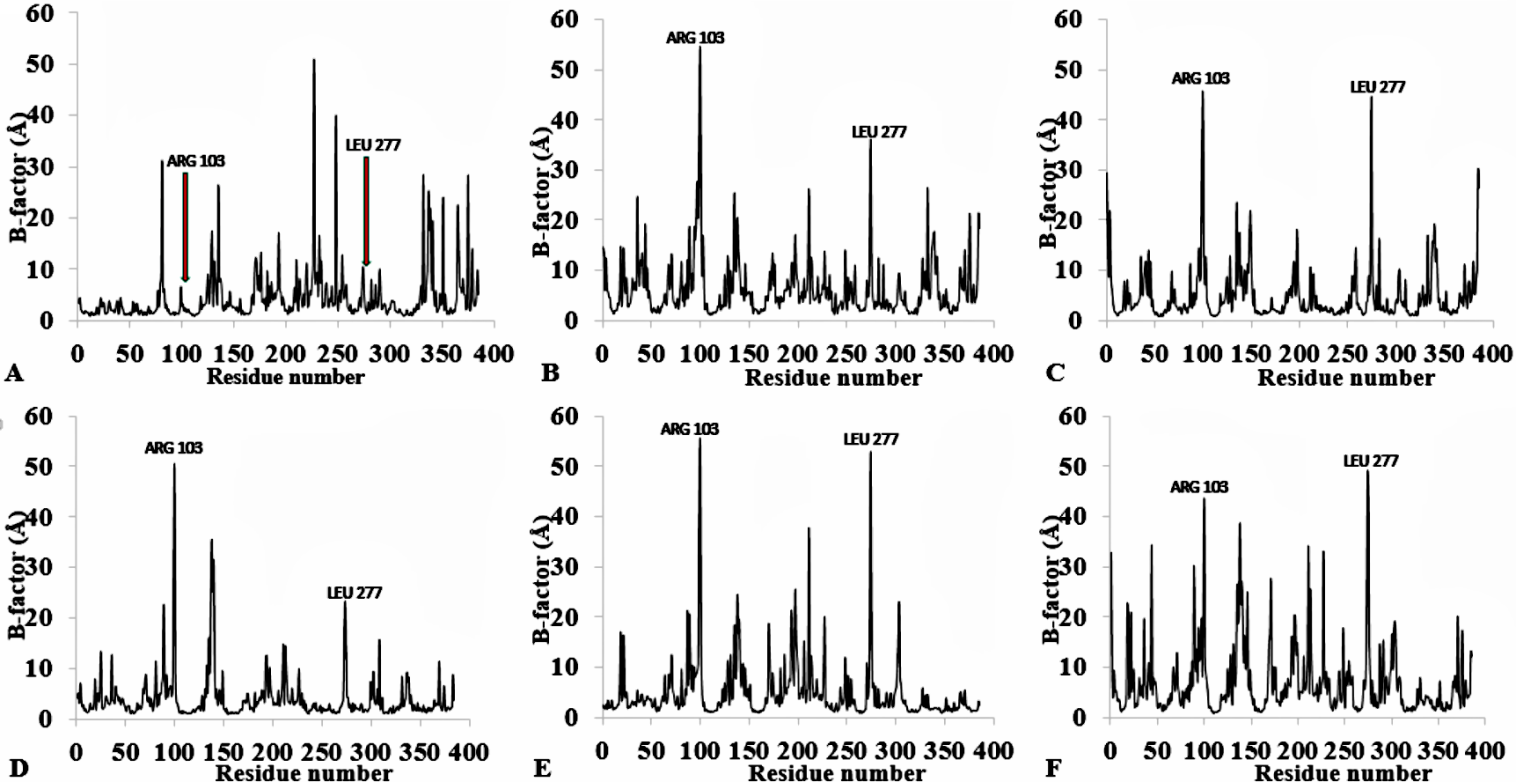
Per-residue B-factor calculated from the last 10 ns of 40 ns simulations for T1 lipase 2DSN. The peaks correspond to the mean average of three replicates simulations at different initial velocities. B-factor were calculated from the root mean square fluctuations (rmsf) in (A) H_2_O, (B) MeOH-H_2_O, (C) BtOH-H_2_O, (D) PrOH-H_2_O, (E) BtOH-H_2_O, (F) PtOH-H_2_O. Higher fluctuations are indicated for Arg 103 and Leu 277.

The flexibility of the residues throughout the time-course was inspected from the calculated measure of the B-factors based on the analysis of the root-mean-square fluctuations (RMSF) (Fig. 3, Fig. S4 and Table 2). The B-factor or temperature factor represents a smearing of atomic electron densities as it governs the equilibrium positions of the atoms due to the thermal motion and positional displacement (Parthasarathy & Murthy, 2000). High thermal motions of residues are proposed targets towards improving the stability of proteins. There is considerable experimental evidence of a strategy that targets residues with high B-factors and has been shown to improve the organic solvent stability of a *Bacillus subtilis* lipase (Reetz et al., 2010). In this regard, the atomic motion of the residues was high in response to decreasing solvent polarity indicating increased flexibility. In particular, the flexibility of residues was more confined to the loop region at the C-terminal in all solvent simulations, with decreasing flexibility and increased rigidity of the N-terminal region in H_2_O, MtOH-H_2_O, EtOH-H_2_O, and PrOH-H_2_O. However, two distinguishable significant fluctuations were by residue Arg103 and Leu277. These residues are positioned on the surface of the protein and exposed directly to the solvent environment. Furthermore, we investigated the unambiguous effect of exposure of the amino acid residues to the solvents and other interactions that could influence the flexibility. We found that the amino acid residues deployed in close proximity to Arg103 were non-aromatic side chain residues. The separation distance between the cation group of Arg103 and the aromatic ring residues is far apart, which could not have allowed Arg103 to experience a favourable cation-*π* interaction, thereby becoming energetically stable and increasing its flexibility (Gallivan & Dougherty, 1999). Similarly, no neighbouring acidic residues for the formation of salt bridges occur to stabilize the loop region of Arg103 to restrict it conformational flexibility. The observed flexibility of Leu277 suggest that it cannot interact strongly with other neighbouring residues that are not hydrophobic. Therefore, the direct exposure of these residues to the solvents triggered their flexibility. Strub et al. (2004) identified mutational substitution to lower the surface area of hydrophobic residues with amino acid arginine, and to significantly enhance the stability of protein in organic solvents. Highly flexible residues could trigger protein unfolding due to their large fluctuation. Moreover, the flexibility can also be used as an indicator to find unstable residues whose cooperative interactions can be reinforced to improve stability (Bae & Phillips, 2005; Joo et al., 2011; Reetz, Carballeira & Vogel, 2006).

**Table 2:** Overall simulation properties, calculated from 40 ns simulations of T1 lipase (2DSN) in different polar solvent mixtures and averaged over all replicates.

	H_2_O			MtOH			EtOH			PrOH			BtOH			PtOH		
	Mean	s.d.	s.e.	Mean	s.d.	s.e.	Mean	s.d.	s.e.	Mean	s.d.	s.e.	Mean	s.d.	s.e.	Mean	s.d.	s.e.
rmsd	0.723	0.03	0.02	1.136	0.07	0.05	1.040	0.11	0.06	1.194	0.04	0.02	1.195	0.09	0.05	1.241	0.05	0.03
B-factor	4.00	0.00	0.00	6.00	1.73	1.00	5.0	1.2	0.7	4.0	1.2	0.7	5.0	3.1	1.7	7.0	2.5	1.5
Rgyr	20.8	0.1	0.0	23.1	0.2	0.1	22.3	2.0	1.0	23.9	0.1	0.1	23.8	0.1	0.1	23.7	0.6	0.4
Lid distance	13.7	0.6	0.3	15.4	1.5	0.9	14.7	1.2	0.70	14.3	0.5	0.3	15.7	0.6	0.3	15.0	0.0	0.0
Lid rmsd	0.60	0.1	0.0	0.97	0.1	0.1	0.95	0.1	0.1	0.80	0.1	0.1	0.97	0.1	0.1	0.81	0.1	0.0
saltbridge	14.0	1.0	0.6	15.6	1.4	0.8	13.6	0.6	0.3	14.9	0.9	0.5	16.9	1.73	1.0	16.6	1.2	0.7
Active site (°)	49.5	0.6	0.3	48.0	1.7	1.0	49.0	2.0	1.2	49.0	3.8	2.2	52.0	4.2	2.4	51.0	1.5	2.4
Hbond	239	6.0	3.5	250	7.0	4.0	241	11.0	6.4	291	34.0	19.5	239	21.8	10.9	245	38.9	13.8
SASA	20272	251	145	35239	1348	778	37229	753	435	41572	2507	1447	39005	1505	869	40188	4688	2707

**Notes.**

Values for simulations of T1 lipase over 40 ns timescale were computed and the following quantities are given: mean, mean value; s.d, standard deviation; s.e. standard error for the following simulation properties; All rmsd of backbone atoms; B-factor calculated from the rmsf of all backbone atoms, *R*gyr, radius of gyration. Lid distance based on the distance between Res Asp175 CA and Res Arg230 CA atom; backbone rmsd for residues Asp175-Arg230 constituting the lid region; number of salt-bridges involving Res Lys Atom NZ with distance <4 from Asp Glu Atom OD and OE, Res Arg Atom NE, NH with distance <3.5 from Asp Glu Atom OD, OE; the bond angle (°) between three Calpha atoms of angle CA Res Ser 113, CA Res Asp 317, CA Res His 358 constituting the active site, while that of the crystallized T1 lipase (2DSN) is 43.16; hydrogen bonds (Hbond) within the solute (amino acid residues).

**Figure 4:**
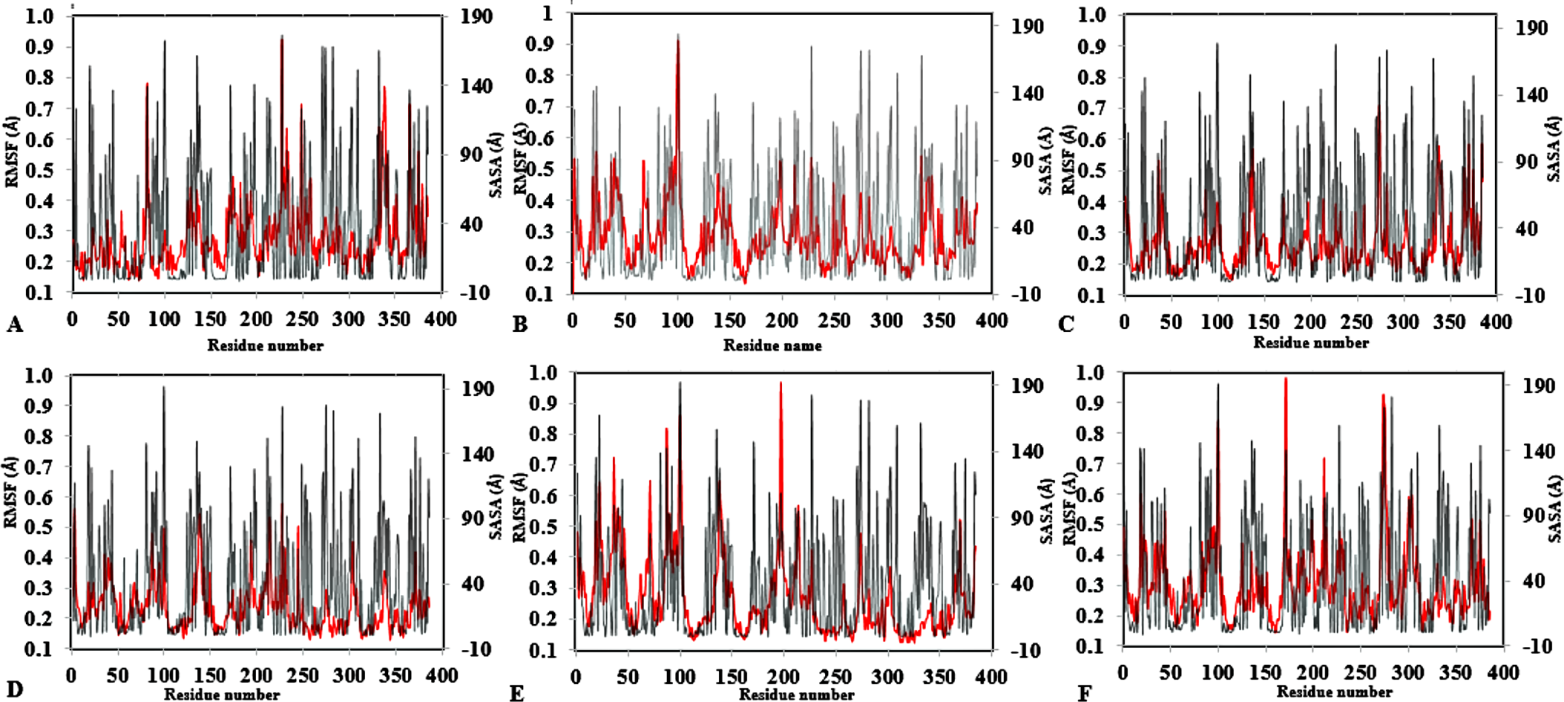
Average RMSF and SASA for each residue in all solvent mixtures (A) H_2_O, (B) MtOH-, (C) EtOH-, (D) PrOH-, (E) BtOH-, and (F) PtOH-H_2_O over the last 150 ps simulations. The red peaks depicts the RMSF per residue, and the gray peaks depicts the SASA per residue of all replicate simulations.

To further validate the relationship between flexibility and surface area/hydrophobicity of residues, the calculated RMSF and SASA from the last 150 ps trajectories were also obtained (Fig. 4). This is important because the RMSF/B-factor of the averaged equilibrium conformation often served as an indicator to estimate flexibility of the protein (a property said to be related to the protein activity) (Li et al., 2010; Yang, Dordick & Garde, 2004). However, to achieve flexibility, the residues are often exposed to the surrounding media thereby increasing their solvent accessibility and contact. In Fig. 4, the higher flexibility indicates that both the hydrophobic and hydrophilic side chains reorient themselves on the protein surface in organic solvents (particularly for Arg103 and Leu277). Similar observations have been reported for other organic solvent media with variations of the solvent composition (Zhu et al., 2012). Usually, solvent-buried residues would have the lower RMSF/B-factor and solvent-exposed residues would have the higher RMSF/B-factor. The correlations in Fig. 4, derived from other reports, describe the correlation between the flexibility of protein and its total SASA, wherein the exposed solvent-accessible surface areas of proteins tend to adopt a more extended and flexible conformation (Marsh & Teichmann, 2011; Gao et al., 2010; Zhang et al., 2009).

The flexibility of static states and flexible motions can be further considered to predict the flexibility changes of local structures with accuracy in different solvent environments (Joo et al., 2011).

### Residue accessible surfaces in structural conformation

**Figure 5:**
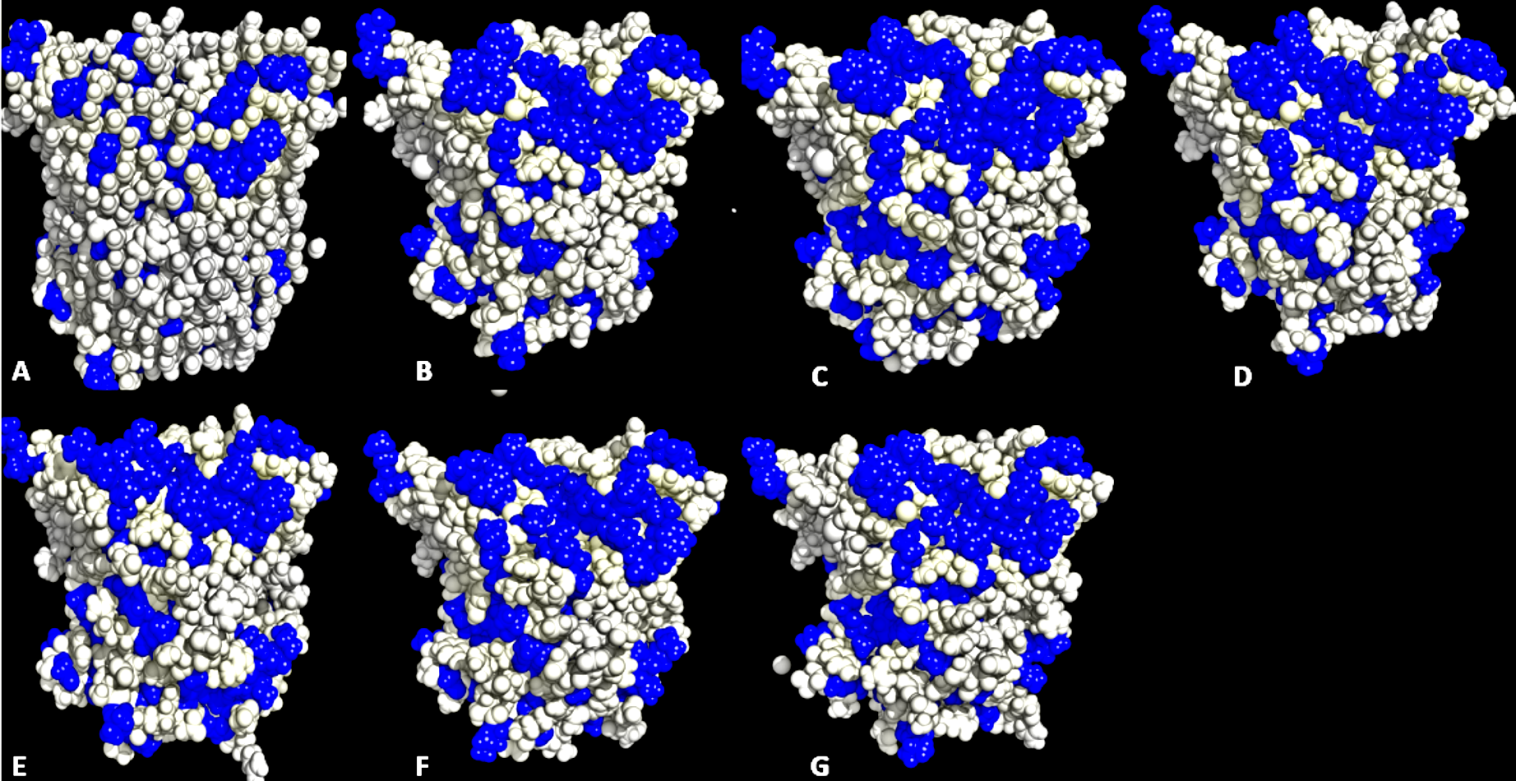
Conformations of exposed hydrophobic residues (blue) of T1 lipase of the last snapshots of 40 ns simulations as compared to (A) crystal structure, (B) H_2_O, (C) MeOH-H_2_O, (D) EtOH-H_2_O, (E) PrOH-H_2_O, (F) BtOH-H_2_O, (G) PtOH-H_2_O solvent mixtures.

Structural movements are a feature that influence the distribution of hydrophobicity of residues. A significant distribution of the hydrophobic surface residues in solvent-exposed positions occurred in all solvent simulations (Fig. 5 and Fig. S5). Factors other than solvents can induce the exposure of hydrophobic residues. Some highly active hydrophobic sites have a tendency for organic solvent denaturation (Chakravorty et al., 2012). T1 lipase crystallized at a lower temperature (20 °C) has its hydrophobic residues tightly packed against each other and profoundly influencing their hydrophobic effects. Organic solvents and temperature-induced fluctuation can have a weakening effect on the hydrophobic core of a protein structure. By the same reasons, organic solvent mixtures and higher temperature simulations induced a dramatic exposure of buried hydrophobic residues. This can significantly allow non-conservative hydrophobic surface residues with higher RMSD and higher hydrophobic area around the hydrophobic patches other than the lid domain to be computationally redesigned to lower the RMSD and hydrophobic area and enhance stability (Park et al., 2013). Substitution of hydrophobic for hydrophilic residues has been demonstrated as a strategy for enhancing protein stability (Pedone et al., 2001; Strub et al., 2004).

**Figure 6:**
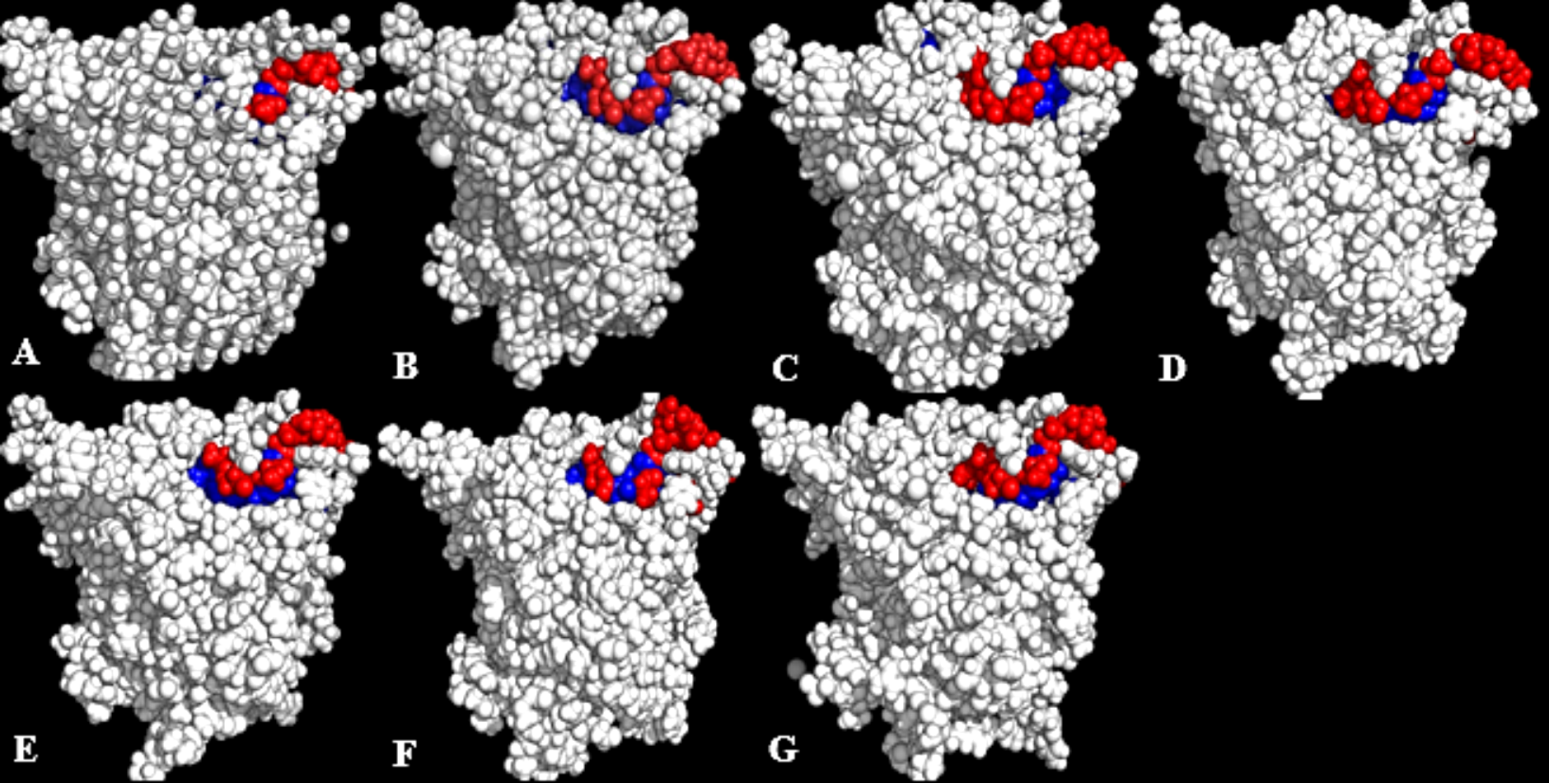
Conformations of exposed (red) and buried (blue) hydrophobic residues constituting the lid regions of T1 lipase of the last snapshots of 40 ns simulations as compared to (A) crystal structure, (B) H_2_O, (C) MtOH-H_2_O, (D) EtOH-H_2_O, (E) PrOH-H_2_O, (F) BtOH-H_2_O, (G) PtOH-H_2_O. At the lid domain, residues with a lower measure of solvent accessible hydrophobic area (<20 Å) were completely buried. Residues with a higher hydrophobic area of the solvent accessible hydrophobic surface were completely exposed.

**Figure 7:**
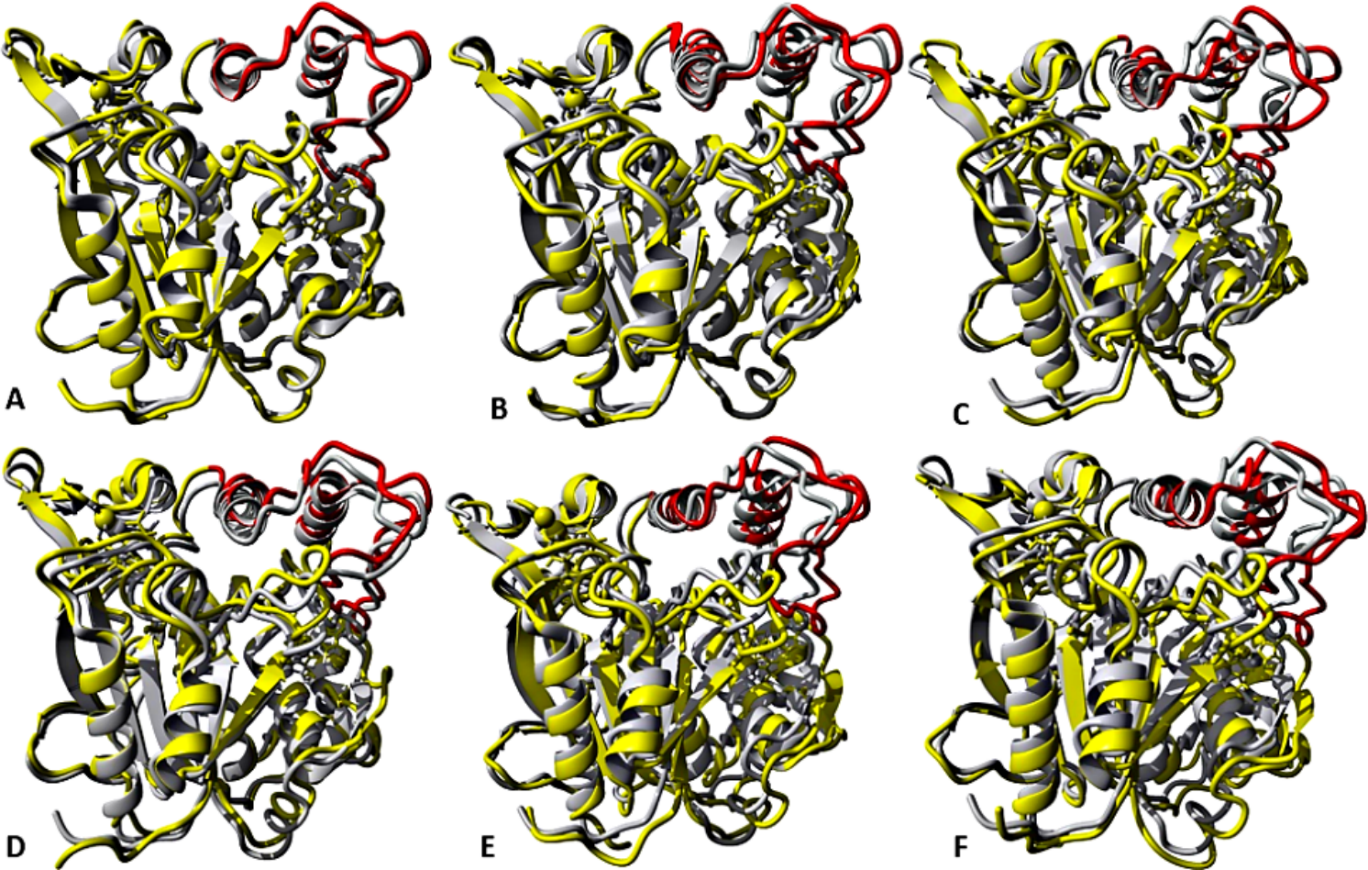
representative structures of the last 40 ns of T1 lipase (yellow) in different solvent mixtures (A) H_2_O, (B) MtOH-H_2_O, (C) EtOH-H_2_O, (D) PrOH-H_2_O, (E) BtOH-H_2_O and (F) PtOH-H_2_O superposed with the reference crystallographic structure (gray). Localized structural differences are observed near the lid domain (red) at the beginning of the helix Asp175 all through the loop to Arg230, which shows a gradual lid opening.

Lipases show a hydrophobic mobile ‘lid’ controlling access to the buried active site, which facilitates their ‘interfacial activation’. In a classical solvent-protein interaction, the lid domain will move away upon contact with solvent molecules, exposing the buried hydrophobic residues to the protein’s surface. The increased hydrophobic area of hydrophobic residues spanning the lid domain (Fig. 6; Table 3) caused a displacement of the lid helical segments in some solvent mixtures. Since conformational changes in proteins towards an activated form is often associated with interfacial activation (Grochulski et al., 1994), hydrophobic residues at the lid are proposed to largely be involved in the conformational changes observed in some solvent mixtures, which greatly expands the nonpolar side of the lid (Fig. 7 & Fig. S6). MD simulations of other lipases indicate that greater conformational change in methanol can destroy the water layer, breaking the hydrophobic packing on the surface and exposing the residues to solvents, which also weakens hydrophobic interactions (Jiang et al., 2014). The exposure of these residues at the lid sometimes can enhance hydrophobic interactions between the enzyme and the solvents as well as interfacial activation (Brzozowski et al., 1991). The high hydrophobic area of Pro^217,198^ (Table 3) will not affect the secondary structure of the protein. This is ultimately due to its preferential location in the accessible turns of proteins and the profound local impacts that a proline can impart to a structure (Lins, Thomas & Brasseur, 2003; Cherukuvada et al., 2005). In addition, its nitrogen backbone is not available for the hydrogen bonding required for helix formation, which renders it immutable to solvent effects as observed in the native T1 structure.

**Table 3:** Total hydrophobic solvent accessible area (Å) of hydrophobic residues of the lid domain from the last structure of 40 ns simulation in solvent mixtures.

Residue	2DSN	H_2_O	MtOH	EtOH	PrOH	BtOH	PtOH
PHE 176	6.23^i^	10.75^i^	20.6^i^	6.30^i^	18.1^i^	9.70^i^	17.00^i^
PHE 180	2.81^i^	4.01^i^	20.0^i^	7.00^i^	3.00^i^	10.20^i^	8.20^i^
PHE 181	0.00^i^	0.00^i^	1.90^i^	4.20^i^	0.40^i^	2.70^i^	0.20^i^
LEU 183	2.39^i^	14.72^i^	35.8^e^	22.50^e^	9.7^i^	17.5^i^	28.70^e^
ALA 186	29.64^e^	32.00^e^	22.6^e^	30.70^e^	24.20^e^	27.20^e^	26.30^e^
VAL 187	0.44^i^	4.88^i^	36.1^e^	21.10^e^	18.30^i^	18.80^i^	16.2^i^
LEU 188	0.00^i^	0.27^i^	9.70^i^	0.80^i^	4.60^i^	1.70^i^	0.20^i^
ALA 190	39.50^e^	53.68^e^	43.3^e^	55.50^e^	49.40^e^	52.60^e^	47.30^e^
ALA 191	5.41^i^	7.41^i^	21.5^e^	8.80^i^	0.50^i^	11.80^i^	6.00^i^
ALA 192	90.77^e^	86.29^e^	80.10^e^	90.00^e^	83.40^e^	74.60^e^	90.80^e^
VAL 193	1.48^i^	3.15^i^	14.40^i^	17.1^i^	2.80^i^	10.60^i^	7.40^i^
ALA 194	40.79^e^	47.42^e^	45.30^e^	48.90^e^	38.40^e^	40.40^e^	45.70^e^
VAL 197	54.73^e^	89.40^e^	65.20^e^	62.10^e^	68.80^e^	83.10^e^	69.90^e^
PRO 198	60.73^e^	62.23^e^	69.6^e^	71.90^e^	70.20^e^	70.80^e^	79.20^e^
VAL 203	11.45^i^	9.83^i^	19.8^i^	31.40^e^	12.30^i^	22.30^e^	13.00^i^
PHE 206	7.58^i^	5.42^i^	11.10^i^	3.50^i^	4.70^i^	4.20^i^	3.80^i^
LEU 208	7.90^i^	8.41^i^	8.80^i^	8.90^i^	3.60^i^	2.30^i^	0.60^i^
GLY 212	63.69^e^	58.28^e^	54.4^e^	51.30^e^	45.00^e^	54.50^e^	46.20^e^
LEU 213	27.59^e^	41.15^e^	33.00^e^	41.90^e^	35.60^e^	25.70^e^	27.50^e^
PRO 217	126.49^e^	129.79^e^	123.9^e^	120.50^e^	131.10^e^	96.30^e^	109.2^e^
GLY 218	63.52^e^	71.13^e^	54.10^e^	59.20^e^	63.50^e^	33.70^e^	40.20^e^
PHE 221	0.00^i^	1.01^i^	1.10^i^	1.50^i^	1.20^i^	0.00^i^	0.60^i^
PHE 225	0.27^i^	0.00^i^	2.60^i^	3.10^i^	3.80^i^	0.40^i^	0.20^i^
LEU 228	0.00^i^	1.70^i^	2.30^i^	7.30^i^	0.00^i^	1.10^i^	0.10^i^

**Notes.**

i Buried.

e exposed.

### Solvent strain effect on conformational stability

**Figure 8:**
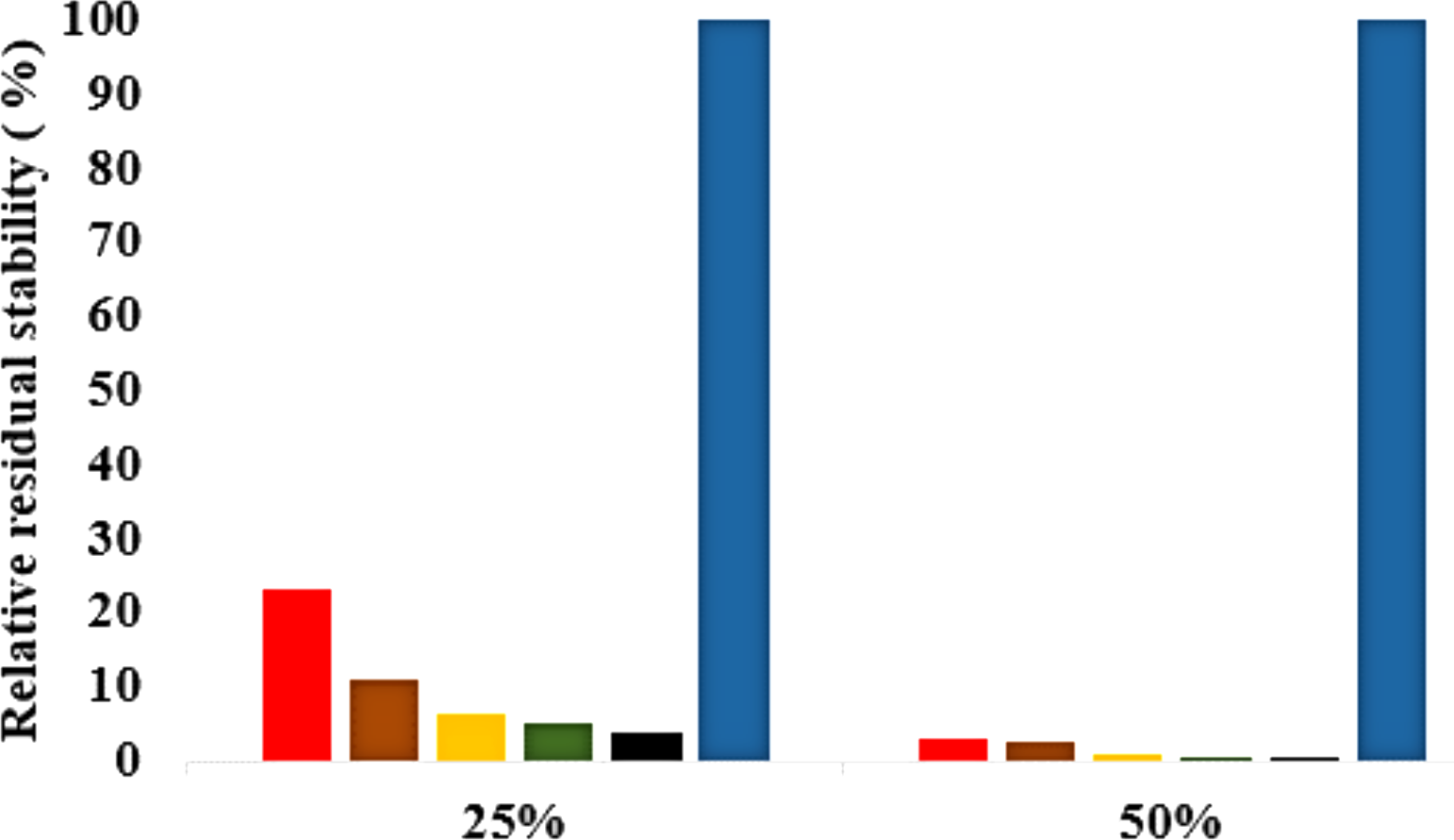
Effects of organic solvent on T1 lipase stability. The lipase activity was determined at 70° C in olive oil emulsion-Glycine-NaOH buffer (pH 9) emulsion as the substrate. After pre-incubation of the lipase at 25% and 50% solvent concentration for 1 h, residual activity was determined according to Kwon & Rhee (1986). The relative residual stability was defined as the activity compared to a native T1 lipase incubated at 50% polar organic solvents and untreated T1 lipase held at 100%. Results are average of three independent experiments. Plots show organic solvent % concentration of treated T1 lipase MtOH *red*, EtOH *brown*, PrOH *yellow*, BtOH *green*, PtOH *black* and untreated T1 lipase *blue* (held at 100%).

**Figure 9:**
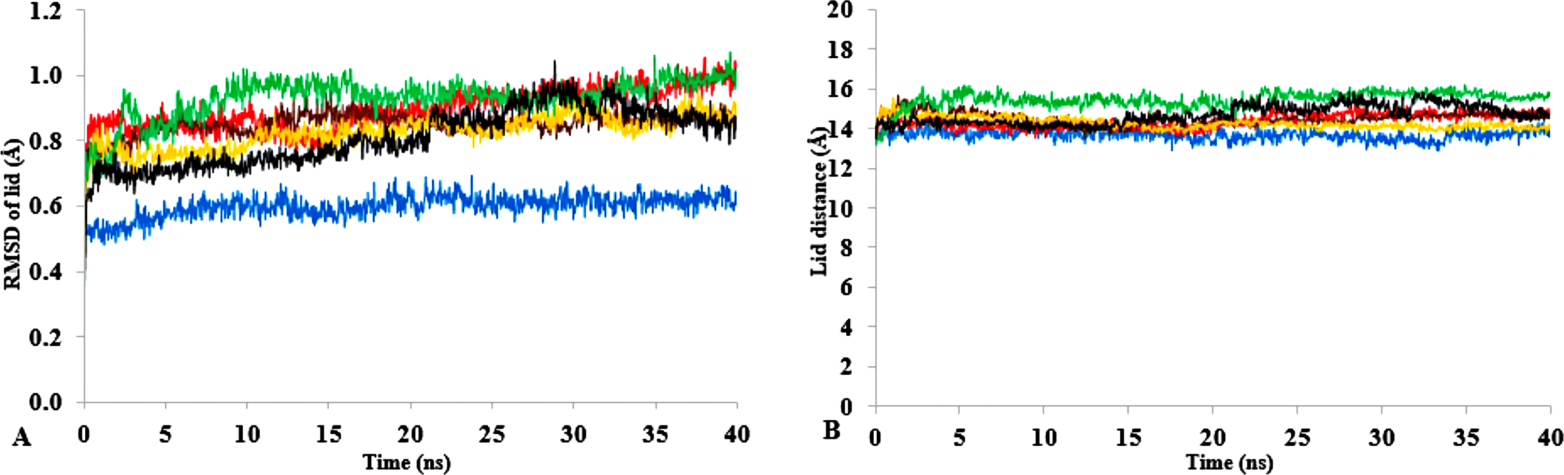
(A) lid domain rmsd calculated from the Cα atoms of residue Asp175-Arg230 in all solvents. (B) Distance of the lid measured between Cα Res Asp175 and Cα Res Arg230 with appreciable increase in distance of lid opening among all solvents. Solvents mixtures are H_2_O (blue), MtOH-H_2_O (red), EtOH-H_2_O (brown), PrOH-H_2_O (yellow), BtOH-H_2_O (green), PtOH-H_2_O (black). All analysis are the mean average of three replicates of 40 ns simulations.

The role of organic solvents on lipase-catalysed reactions in liquid organic media can be understood based on the competitive inhibitory character of solvents, change in conformation and substrate solubility (Graber et al., 2007). Experimentally, the effect of five polar solvents in terms of the measured residual activity had a marked effect on both enzyme stability and activity. If the lipase stability in the presence of polar organic solvents is considered, the destabilizing effect of these solvents is ultimately related to their decreasing polarity and increasing chain length (Fig. 8). Following molecular simulations in the same solvent conditions, analysis of the last structures revealed structural movements of the lid helices involved in conformational rearrangements of some secondary structure elements of the α6-loop-α7 helix (Fig. 7 & Fig. S6). The movements are more significant in all solvent mixtures in contrast to the degree of movement in the H_2_O solvent. Comparisons of the resulting conformational changes of the lid show that the fluctuations seem to trigger the displacement of the lid domain and also correlate with the rms deviations and increased distance between residue Asp175 and Arg230 of the lid (Figs. 9A, 9B & Figs. S7, S8). It is not surprising to observe a gradual displacement of the helical conformation of the lid domain in MtOH-H_2_O solvent mixtures because methanol has also been shown to favour induced conformational transitions in a number of protein folding studies (Babu, Moradian & Douglas, 2001). In fact, these transitions have long been demonstrated in other polar solvents in which they are implicated in the disruption of rigid tertiary structures in protein molecules with the expansion of the helical conformation (Uversky et al., 1997). Although hydrocarbons have not been reported to inhibit enzyme activity experimentally, simulations of T1 lipase in water-octane mixtures strongly suggested otherwise. Large structural rearrangement of the lid domain was caused by the interaction between the hydrophobic residues of the lid with octane. However, removal of octane from the system restored the enzyme conformational state (Graber et al., 2007; Abdul Rahman et al., 2012). High activation energy barriers also control the conformational transition stability of enzymes in organic solvents, whereby the polarity of solvents seems to impact the structural integrity of proteins in different ways (Kovacs et al., 1995; Liu & Hsu, 2003).

### Network of interactions and contributions to stability

**Figure 10:**
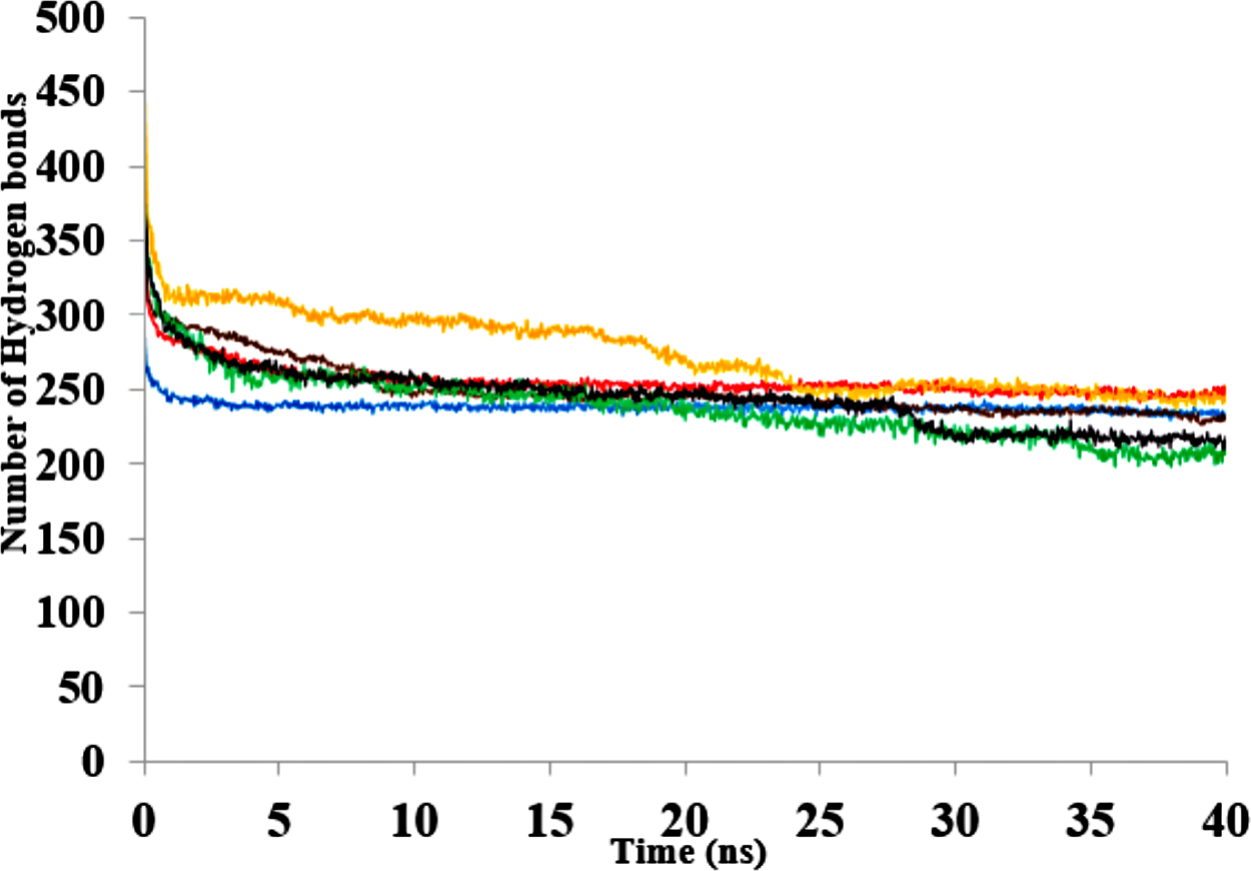
The number of hydrogen bonds within the solute as a function of the H-bond-acceptor distance in Å  of 40 ns simulations. Solvent mixtures are H_2_O (blue), MtOH-H_2_O (red), EtOH-H_2_O (brown), PrOH-H_2_O (yellow), BtOH-H_2_O (green), PtOH-H_2_O (black).

The three-dimensional structure of a protein generally achieves a certain degree of packing and stability due in part to complementary weak interactions. Hydrogen bonding, electrostatic and hydrophobic interactions, and salt bridges all play roles in protein folding and establishing its final structure (Serdakowski & Dordick, 2008; Gallivan & Dougherty, 1999). Polar solvents are strongly competitive with intramolecular hydrogen bonds and are a major cause of disruption to structural and dynamical interactions between protein atoms (Knubovets, Osterhout & Klibanov, 1999). Interactions were analysed in the presence of organic solvent mixtures (Fig. 10A & Fig. S9). It is worth noting that the role of hydrogen bonds in terms of folding and stability of proteins does not necessarily lead to any ordered structural classification and thus a systematic investigation could be difficult (Sanjeev & Vishveshwara, 2002). The analysed hydrogen bonds revealed differences between the numbers of additional hydrogen bonds in all solvents. The hydrogen bonds remained well preserved in BtOH-H_2_O-H_2_O, EtOH- H_2_O and PrOH-H_2_O in the last 10 ns and decreased for BtOH-H_2_O and PtOH- H_2_O solvents. The decreased number of hydrogen bonds was not in response to the solvent polarity throughout the simulations. An ordered secondary structure can only be obtained where there is a large hydrogen bond formation. The introduction of stabilizing internal bonds also minimizes the solvent strain effect limiting aggregation and irreversible inactivation of an unfolded protein in a nonaqueous environment (Arnold, 1990). Hydrogen bonds and other interactions are characterized by the reorientation of the polar amino acid side chains making the protein rigid and compact in an organic solvent (Khabiri et al., 2013; Yang, Dordick & Garde, 2004). Disruption of these interactions could lead to reduced flexibility of residues. This is often due to the decreasing water-mediated network resulting from the exchange of water molecules with solvent molecules at the protein surface (Trodler & Pleiss, 2008; Zheng & Ornstein, 1996).

The contribution of hydrophobic interactions depends on how many nonpolar groups are buried during folding of a protein (Pace et al., 2012). Hydrophobic interactions stabilizing the protein structure remain weakened with increasing carbon atoms of polar solvents (Table 4). The reduced amount of interactions highlights the general role of hydrophobic interactions between a target protein and the surrounding environment. The hydrophobic and hydrophilic properties of the organic solvent in a protein environment as well as exposed surface residues of the protein govern the type of interactions that are formed between protein and solvent molecules (Patargias, Harris & Harding, 2010). Previous results from MD simulations have also elaborated on how methanol strengthens hydrogen bonding and weakens hydrophobic interactions in proteins (Hwang et al., 2011).

**Table 4:** Total hydrophobic interactions calculated from the last structure of 40 ns simulation in co-solvent mixtures.

Solvents	Protein	Protein-Hetgroup
T1 (2DSN)	810	–
H_2_O	789 12.7 8.9	–
MtOH	747 14.0 9.9	260 10.6 7.7
EtOH	729 4.0 2.3	232 7.8 5.7
PrOH	728 6.3 4.2	230 7.0 5.0
BtOH	737 0.7 0.5	205 1.2 0.6
PtOH	722 3.5 2.5	113 2.1 1.4

**Notes.**

Data are mean, standard deviation and standard error respectively. The amounts of hydrophobic interactions from the last structures were calculated for interaction distances below 5 Å  with all solvent molecules treated as heterogeneous group (Hetgroup).

### Geometric orientation changes in active site residues, Ca^2+^ and Zn^2+^ as it affects protein stability

The overall rms deviation of the structure does not capture the details of any implied global conformational changes to the active site or metal cofactors. Therefore, the distances of the proximal residues holding the protein metal ions in place were analysed (Table 5). Distances and bond angles between residues differed slightly from the initial structure 2DSN. The metal ions and bond angle appear to drift from their original position as shown by the increase of their distance of separation. The movements of the coordinating residues of the metal cofactors can allow accessibility to the metal binding pocket, which is also important in the interfacial activation of lipases. Molecular simulations have also revealed the geometric displacement of these metal cofactors and the coordinating residues cause major structural transitions in lipases (Invernizzi et al., 2009).

**Table 5:** Some key geometric parameters of coordination position of Ca^2+^ and Zn^2+^ metal ions obtained from the last structure of 40 ns simulations of T1 lipase in polar co-solvents.

Solvents	Ca^2+^						Zn ^2+^					
	Residue/metal ion distance (Å)						Residue/metal ion distance (Å)					
	Gly286	Glu360	Asp365	Pro366	Metal ion	Bond (°)	Asp61	His81	His87	Asp238	Metal ion	Bond (°)
2DSN	2.2	2.32	2.44	2.37			2.7	2.1	2.1	2.1		72.03
H_2_O	2.2 0.0 0.0	2.3 0.0 0.0	2.4 0.0 0.0	2.4 0.0 0.0	1.0 0.0 0.0	42 0.1 0.1	2.7 0.0 0.0	2.1 0.0 0.0	2.1 0.0 0.0	2.1 0.0 0.0	0.3 0.1 0.1	79 1.2 0.8
MtOH	2.2 0.0 0.0	2.3 0.0 0.0	2.4 0.0 0.0	2.4 0.0 0.0	2.0 0.0 0.0	42 2.2 1.3	2.7 0.0 0.0	2.1 0.0 0.0	2.1 0.0 0.0	2.1 0.0 0.0	1.2 0.2 0.1	76 0.3 0.2
EtOH	2.2 0.0 0.0	2.3 0.0 0.0	2.4 0.0 0.0	2.4 0.0 0.0	1.5 0.1 0.1	43 1.0 0.6	2.7 0.0 0.0	2.1 0.0 0.0	2.1 0.0 0.0	2.1 0.0 0.0	1.3 0.2 0.1	73 0.9 0.5
PrOH	2.2 0.0 0.0	2.3 0.0 0.0	2.4 0.0 0.0	2.4 0.0 0.0	1.9 0.4 0.3	44 0.9 0.6	2.7 0.0 0.0	2.1 0.0 0.0	2.1 0.0 0.0	2.1 0.0 0.0	1.4 0.5 0.4	74 1.8 1.3
BtOH	2.2 0.0 0.0	2.3 0.0 0.0	2.4 0.0 0.0	2.4 0.0 0.0	1.8 0.3 0.3	46 2.7 1.6	2.7 0.0 0.0	2.1 0.0 0.0	2.1 0.0 0.0	2.1 0.0 0.0	0.9 0.2 0.1	73 0.2 0.1
PtOH	2.2 0.0 0.0	2.3 0.0 0.0	2.4 0.0 0.0	2.3 0.0 0.0	1.8 0.2 0.1	43 0.6 0.3	2.7 0.0 0.0	2.1 0.0 0.0	2.1 0.0 0.0	2.1 0.0 0.0	1.6 0.1 0.1	77 1.3 0.9

**Notes.**

Bond angle (°) and metal ion distances represents data from the superposed last structures of 0 ns and 40 ns simulations. Residue distance of atoms O-Gly268 →OD2-Asp365 →O-Pro366 →OE2-Glu360 and CG-Asp61 →HE-His81 →NE2-His87 →OD2Asp238 were measured from the metal ions Ca^2+^ and Zn ^2+^ positions of last the structure, respectively.

**Figure 11:**
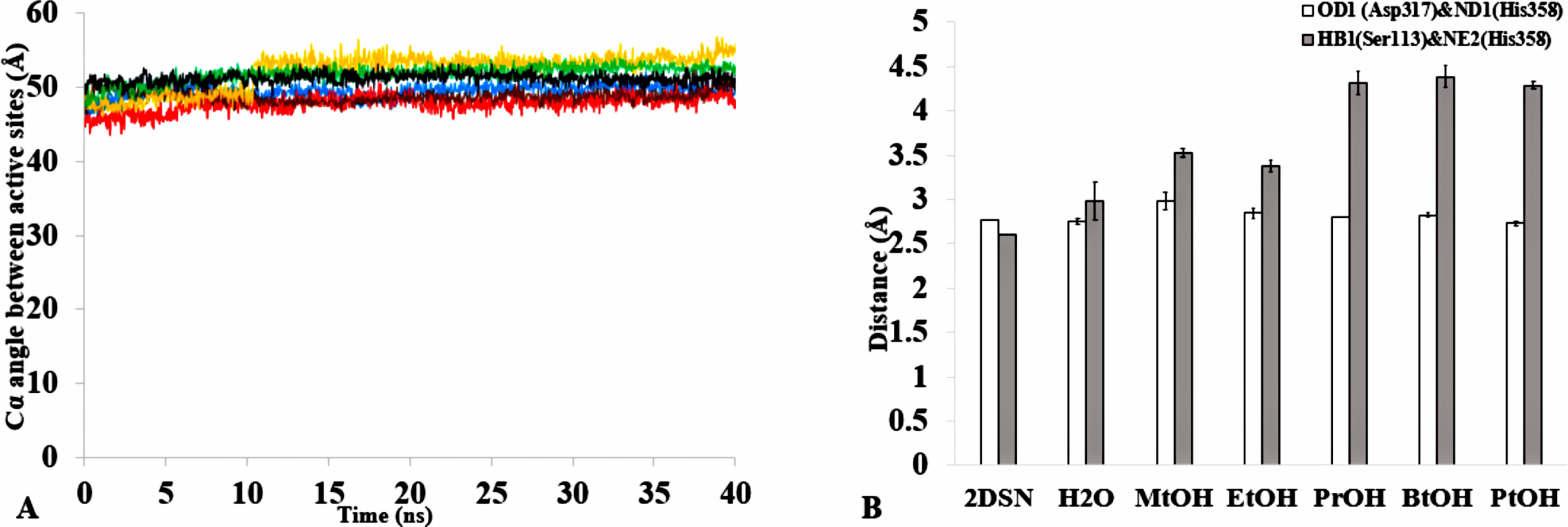
(A) The angle between the active site residue Cα atoms of Ser113, Asp 317 and His358 in all solvents environments of H_2_O (blue), MtOH-H_2_O (red), EtOH-H_2_O (brown), PrOH-H_2_O (yellow), BtOH-H_2_O (green), and PtOH-H_2_O (black). (B) The distance between the active site residues of the last structure from the solvent mixtures environments between OD2-Asp317 and ND1-His358 (bars in white) is conserved, allowing the formation of Hbond. However, the distance is not conserved between HB1-Ser113 and NE2-His358 (bars in gray) in the organic solvent mixtures. One hydrogen bond is considered to be presented if both the distance between the hydrogen atom acceptor and hydrogen atom donor is <3.5. All analysis are the mean average of three replicates of 40 ns simulations.

The formation of hydrogen bonds is central to the transition state in an ester synthesis mediated by the Ser113 acting as a nucleophile promoted by His358, which accepts a proton from Ser113. Although the angle of the alpha carbons of the active sites residues was not preserved in the solvents when compared with the crystallized T1 lipase (2DSN) with a bond angle of 43.16 Å, the distance between Asp317 and His358 of the active site in the last structure was preserved (Figs. 11A,11B, Fig. S10 & Table 2). However, Ser113 and His358 had more conserved distances in H_2_O. The conserved distance in MtOH- H_2_O and EtOH-H_2_O is sufficient to an extent to allow the protonation of His358 by Ser113, which can promote binding and catalysis. This accounts for the higher activity observed for MtOH-H_2_O and EtOH- H_2_O in Fig. 8 in contrast to other solvents. This observation suggests that the solvents influenced the minimum distance between these residues, which is crucial in the formation of H-bonds that play significant stabilization functions in the transition state of the lipase reaction (Carlqvist et al., 2003). Similarly, widening and stability of the active site cavity mediated by the hydrogen bonds may also affect proper positioning and geometric orientation of the backbone atoms of residues, which are crucial for the effective catalytic activity in polar solvents.

**Figure 12:**
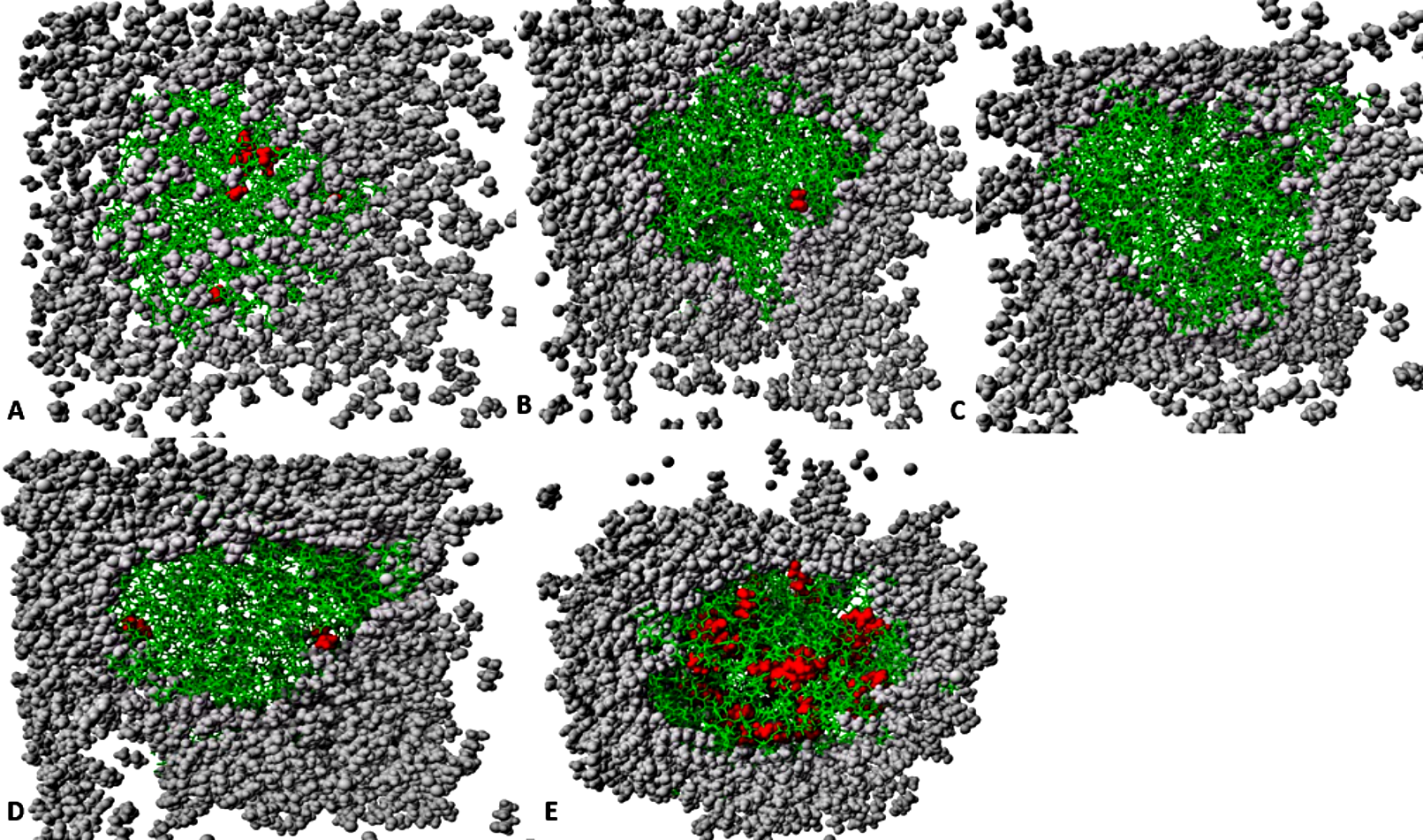
The penetration of organic solvent molecules into the protein as revealed from last trajectory of 40 ns simulations in (A) MtOH-H_2_O, (B) EtOH-H_2_O, (C) PrOH-H_2_O, (D) BtOH-H_2_O, and (E) PtOH-H_2_O. The organic solvent molecules that penetrated into the protein core are in red spheres, while other molecules are represented in gray spheres and protein structure represented in green sticks. Pentanol solvent molecules had higher penetration as compared to other organic molecules.

### Solvent distribution around proteins and the penetration effect on stability

The number of organic solvent molecules introduced into each simulation cell varied (Table 1). Water molecules will normally hydrate ionic and polar sites on the surface of the protein, which creates an exposure of the enzyme surface that is accessible by the organic solvents into the protein core (Yang, Dordick & Garde, 2004). We evaluated the consequences of these effects and found that indeed solvent molecules populate approximately the same regions of the protein surface in the last 40 ns structure (Fig. 12). Interestingly, it can be observed that the number of solvent molecules that migrated slightly down to the core of the protein were considerably higher in pentanol than other solvent simulations. The penetration of organic solvent molecules could have a strong interaction with surrounding residues. Organic solvent molecules appeared arranged in spatial proximity to the protein surface, which could strongly influence the protein stability. However, the size of the organic solvent molecules does not appear to govern solvent penetration. Clustered networks of the solvent molecules around the immediate vicinity of the enzyme-surrounding surface can elicit remarkable effects with the tendency to establish a solvation effect on the residues. Similarly, significant penetration of more PtOH solvent molecules further into close proximity to the active sites and the surrounding residues (despite its size) is governed by its decreased polarity, and will ultimately alter compositions of electrostatic interactions, increase the hydrophobic area and play a prominent role in reduced secondary structure contents. In fact, solvent penetration not only constrains the stability of the protein but also causes dramatic effects on the overall folding stability and dynamics. This is in good agreement with simulations in polar organic solvent-water mixtures in which the accumulation of ethanol molecules in the core of protein causes large conformational changes destroying its structure (Lousa, Baptista & Soares, 2012).

**Figure 13:**
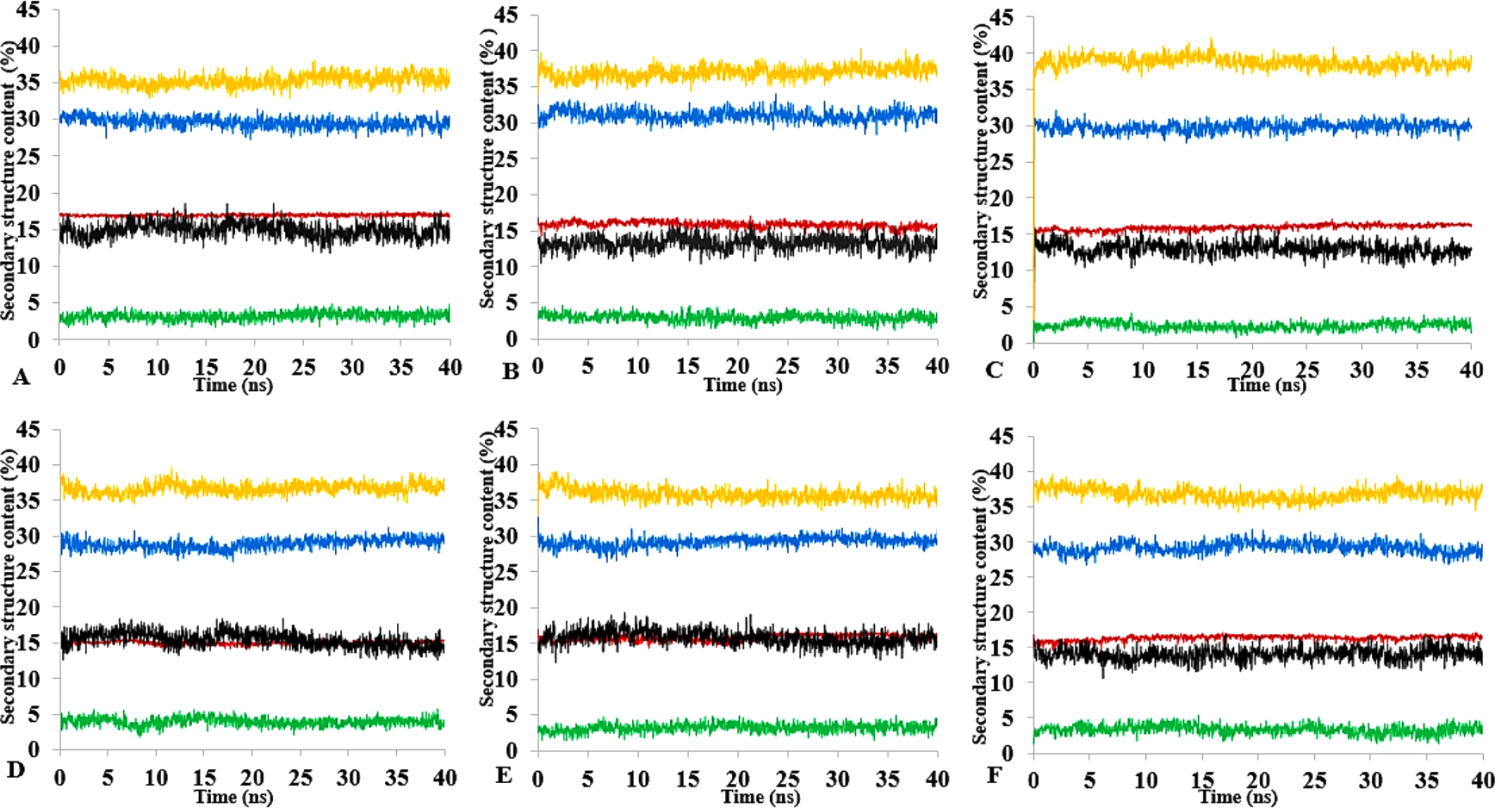
Mean average of replicate 40 ns simulations of preserved secondary structure assignment of T1 lipase in H_2_O (A), MtOH-H_2_O (B), EtOH-H_2_O (C), PrOH-H_2_O (D), BtOH-H_2_O (E), PtOH-H_2_O (F). Increase in coil (yellow) and decrease in sheet (red) occurred in some solvents. The solvents did not have a significant influence on the turn (black), helix (blue) and 3–10 helix (green).

### Secondary structure content analysis

The amino acid residues and the protein structure ultimately share a relationship that characterizes the secondary structure assignment of an entire protein. The preserved secondary structure assignment analysis is presented in Fig. 13 & Fig. S11, and the residue secondary structure structural analysis is presented in Fig. S12. There were alterations to the structure and dynamics of the T1 lipase in all environments, mostly to the helices and coils. The helix and helix 3–10 were preserved in all solvent mixtures implying preserved structural flexibility and rigidity (Khabiri et al., 2013). Variations in residue structural elements (Fig. S12) is a common effect of such polar solvents on lipases, whereby residue positions at the protein surface expose them to such rearrangements as observed in the α-helices contents of a *Candida albican* lipase (Li et al., 2010). It follows then that most protein structures have the propensity to undergo a conformational transition in certain organic solvent-induced environments (Hirota, Mizuno & Goto, 1997; Shiraki, Nishikawa & Goto, 1995).

**Figure 14:**
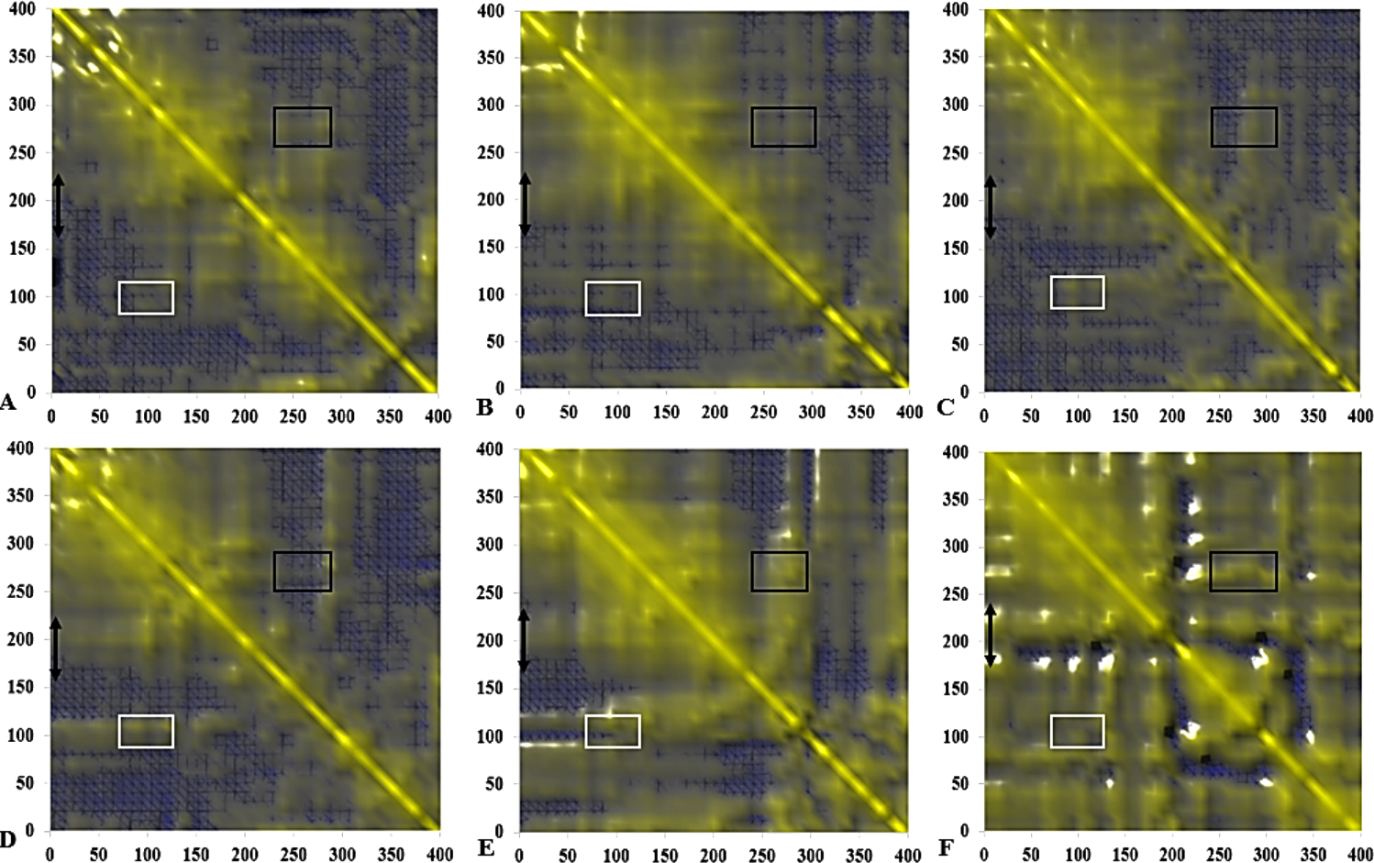
Calculated dynamical cross-correlations map for T1 lipase over the last 10 ns of 40 ns simulations in (A) H_2_O, (B) MtOH-H_2_O, (C) EtOH-H_2_O, (D) PrOH-H_2_O, (E) BtOH-H_2_O, (F) PtOH-H_2_O solvent mixtures. The DCCM matrix is visualized automatically with colours ranging from blue (−1, fully anti-correlated) to yellow (+1, fully correlated) with the zero level (0, not correlated) indicated by the blue wire-frame grid. Correlated movements between residues 175–230 to other residue is highlighted with a black double arrow. Regions comprising correlated movements of residues Arg103 to Leu277 are shown with white and black boxes, respectively. All correlation analysis were considered from the *y*-axis across the maps.

### Dynamic correlated and concerted motions of protein residues

The functional flexibility of proteins and the relatively concerted motions between groups of protein atoms, could determine large-scale conformational transitions and provide a wide range of protein conformations. This is important in understanding the biological functions of proteins based on the co-operative and coherent movement of amino acid residues (Arcangeli, Bizzarri & Cannistraro, 2001). We analysed the collective dynamic characteristics exhibited by the protein in the different solvent mixtures based on the DCCM, which reports the time-correlated motions of atomic pairs (Fig. 14). Generally, positive correlations involve neighbouring groups, which move together, and anti-correlated motions are of particular interest (Luo & Bruice, 2002). In all maps, region Asp175-Arg230 spanning the lid has strongly correlated movements to residues Ile107-Gln114 (which comprise the helix and sheet on which the active site Ser113 is located) and perfectly correlated to the loop residues Ala109-Glu219 (along the diagonal) connecting the α6 and α7 helix of the lid in all solvents. The helix region of Met287-Ser301 shielding the active site residues Asp317 and His358 were strongly correlated to the lid region in BtOH-H_2_O and PtOH- H_2_O and the region on which the active site Ser113 is located (Ile107-Gln114) in all solvent mixture systems. The region of the front part of the active site (His14-Ala52) has some weak anticorrelated motions with the lid in H_2_O and correlated movements in MtOH-, EtOH-, PrOH-, and BtOH-H_2_O with a strongly correlated motion in PtOH-H_2_O. Strongly correlated motions between residues Ala52-Pro95 comprising two helices below the lid occurred in all solvent mixture systems. Checking residues Arg103 and Leu277 in all systems indicates they were weakly correlated in H_2_O, MtOH-, EtOH- and PrOH-H_2_O, but strongly correlated with surrounding residues in the BtOH- H_2_O and PtOH-H_2_O solvent mixture systems.

The negatively correlated motions of DCCM occurs mostly among residues that are far apart in the secondary structure of the protein. Correlation patterns within the protein are more pronounced in an approximately concerted fashion, but the correlation coefficients obtained from the DCCM method do not bear any information about the magnitude of the motions. It does, however, highlight the consistency with simulated B-factors corresponding to the increasing chain length of polar solvents.

## Conclusions

Molecular dynamic simulations of proteins in organic solvents have provided insights into the molecular events a protein undergoes in both aqueous and nonaqueous microenvironments and how they will dynamically interact with flexible protein targets. In this study, molecular dynamic simulations were used to study the conformational changes and stability of T1 lipase in increasing carbon length polar solvents. The simulations revealed conformational displacement of the secondary structure elements. The interactions of the solvent molecules with the protein revealed that polarity of organic solvents play a major role in exposing buried hydrophobic residues with concomitant disruptions of their interactions. The stability of the protein could be maintained in methanol and ethanol solvents in contrast to other solvents. It can be inferred then that MtOH-H_2_O, EtOH-H_2_O mixtures will likely not affect substantial parts of the enzyme microenvironment for substrate binding and activity, provided the active site loop dynamics or position of solvent affecting residues are conformationally undisrupted. It is apparent that organic solvents could affect the transition state in the lipase reaction when the interactions of the active pocket residues are restricted. The displacement of the lid region showed a double lid movement for T1 lipase induced by the solvent molecules. This displacement was triggered by the conformational rearrangements of the Asp175-Arg230 induced by solvent molecules. DCCM and the B-factor reveal strong motions of the residues and higher flexibility in BtOH-H_2_O and PtOH-H_2_O correlated to surrounding residues. Upon further observation we found that penetration of solvent molecules was a response to the solvent decreasing polarity. Such penetration contributed to the strain effect and global conformational changes of the protein. The large hydrophobic surface area of some residues exposed them to the solvents and caused stronger interactions, making the protein surface permeable to the solvent molecules. Our simulation studies suggest that these could render loss of cooperative interactions and ultimately make the protein structurally more floppy. The resultant enhanced fluctuations in some solvents results in the orientation changes of the backbone atoms of the residues, which could lead to difficulties in obtaining functional and biochemical differences in experimental feedback about the dynamics of protein in nonaqueous media. This study provides an opportunity to understand the structural effects of length variations in polar organic solvents on the conformation and stability of T1 lipase.

##  Supplemental Information

10.7717/peerj.3341/supp-1 Figure S1 The time dependence of the root mean square deviations (rmsd) of backbone atoms for the 40 ns simulations of T1 lipase as a function of simulation conditions conducted in three replicates All replicate were run at different initial starting velocities determined based on the RandomSeed whereby the atom velocities are assigned randomly, using the built-in random number generator. Plots show first replicate (black), second (blue) and third (red) in (A) H_2_O (B) MeOH-H_2_O (C) EtOH-H_2_O (D) PrOH-H_2_O (E) BtOH-H_2_O (F) PtOH-H_2_O.

10.7717/peerj.3341/supp-2 Figure S2 The time dependence of the mean average of replicates of the Solvent accessible surface area (SASA) Replicates were generated for (A) H_2_O (B) MeOH- H_2_O (C) EtOH-H_2_O (D) PrOH-H_2_O (E) BtOH-H_2_O (F) PtOH-H_2_O. Plots show first replicate (red), second (black) and third (blue).

10.7717/peerj.3341/supp-3 Figure S3 The time dependence of the mean average of replicates for the radius of gyration Radius of gyration which shows the same level of compactness among solvents compared to H_2_O. Both properties are calculated from mean average of three replicates of 40 ns simulations for (A) H_2_O (B) MeOH-H_2_O (C) EtOH-H_2_O (D) PrOH-H_2_O (E) BtOH-H_2_O (F) PtOH-H_2_O. Plots show first replicate (red), second (black) and third (blue).

10.7717/peerj.3341/supp-4 Figure S4 The time dependence of the B-factor (rmsf) of backbone atoms for the last 10 ns of 40 ns simulations B factor of T1 lipase as a function of simulation conditions in three replicates for (A) H_2_O (B) MeOH-H_2_O (C) EtOH-H_2_O (D) PrOH-H_2_O (E) BtOH-H_2_O (F) PtOH-H_2_O. Plots show first replicate (red), second (black) and third (blue).

10.7717/peerj.3341/supp-5 Figure S5 Back view of conformations of exposed hydrophobic residues Exposed hydrophobic residues (blue) of T1 lipase of the last snapshots of 40 ns simulations as compared to (A) crystal structure (B) H_2_O (C) MtOH-H_2_O (D) EtOH-H_2_O (E) PrOH-H_2_O (F) BtOH-H_2_O (G) PtOH-H_2_O solvent mixtures. Hydrophobic residues were mapped using Open-Source PyMOL 1.7.2.1 (DeLano, 2002).

10.7717/peerj.3341/supp-6 Figure S6 Representative structures of last 40 ns of T1 lipase in different solvent mixtures (A) H_2_O (B) MtOH-H_2_O (C) EtOH-H_2_O (D) PrOH-H_2_O (E) BtOH-H_2_O (F) PtOH-H_2_O superposed with the reference crystallo Localized structural differences are observed near the lid domain (yellow), at the beginning of the helix Asp175 all through the loop to Arg230 which shows a gradual lid opening. The first replicate is presented in Fig. 7 and other replicates are colored in red and blue for respective solvents. Water (A) replicates are superposed together with no structural differences.

10.7717/peerj.3341/supp-7 Figure S7 Root mean square (RMSD) of the lid domain of all simulations Lid domain rmsd calculated from the C *α* atoms of residue Asp175-Arg230 in all solvents. Solvents mixtures are H_2_O (A), MtOH-H_2_O (B), EtOH-H_2_O (C), PrOH- H_2_O (D), BtOH- H_2_O (E), PtOH- H_2_O (F). Plots show first replicate (red), second (black) and third (blue).

10.7717/peerj.3341/supp-8 Figure S8 Distance of lid measured in Åfor all simulations Distance of the lid measured between C *α* Res Asp175 and C *α* Res Arg230 with appreciable increase in distance of lid among all solvents. Solvents mixtures are H_2_O (A), MtOH- H_2_O (B), EtOH- H_2_O (C), PrOH- H_2_O (D), BtOH- H_2_O (E), PtOH- H_2_O (F). Plots show first replicate (red), second (black) and third (blue).

10.7717/peerj.3341/supp-9 Figure S9 Intraprotein hydrogen bonds occurrence within the protein residues in all simulations The number of hydrogen bonds within the solute as a function of the H-bond-acceptor distance in Åof 40 ns simulations. Solvent mixtures are H_2_O (A), MtOH-H_2_O (B), EtOH-H_2_O (C), PrOH-H_2_O (D), BtOH-H_2_O (E), PtOH-H_2_O (F). Plots show first replicate (red), second (black) and third (blue).

10.7717/peerj.3341/supp-10 Figure S10 Geometric bond angle of the tetrahedral intermediate packing of the active site pocket The angle between the active site residue C *α* atoms of Ser113, Asp 317 and His358 of H_2_O (A), MtOH-H_2_O (B), EtOH-H_2_O (C), PrOH-H_2_O (D), BtOH-H_2_O (E), and PtOH- H_2_O (F). Plots show first replicate (red), second (black) and third (blue).

10.7717/peerj.3341/supp-11 Figure S11 Preserved secondary structural contents The secondary structural analysis of T1 lipase over 40 ns simulations in H_2_O (A), MtOH- (B), EtOH- (C), PrOH- (D), BtOH- (E) and PtOH-H_2_O (F) solvent mixtures. All structural elements are shown in blue (helix), red (sheet), black (turn), yellow (coil), green (3–10 helix).

10.7717/peerj.3341/supp-12 Figure S12 Residue secondary structure content of all simulations

## Additional Information and Declarations

### Data Availability

The following information was supplied regarding data availability: Jonathan, Maiangwa (2017): Raw data set for the manuscript PeerJ 11801 on Lid opening and conformational stability of T1 lipase is mediated by increasing chain length polar solvents. Figshare.  https://doi.org/10.6084/m9.figshare.4924013.v2.
