# Supplementary figures and images for: Lid opening and conformational stability of T1 Lipase is mediated by increasing chain length polar solvents
Source: PeerJ. 2017 May 18;5:e3341. doi: 10.7717/peerj.3341 (PMC5438581; doi:10.7717/peerj.3341)

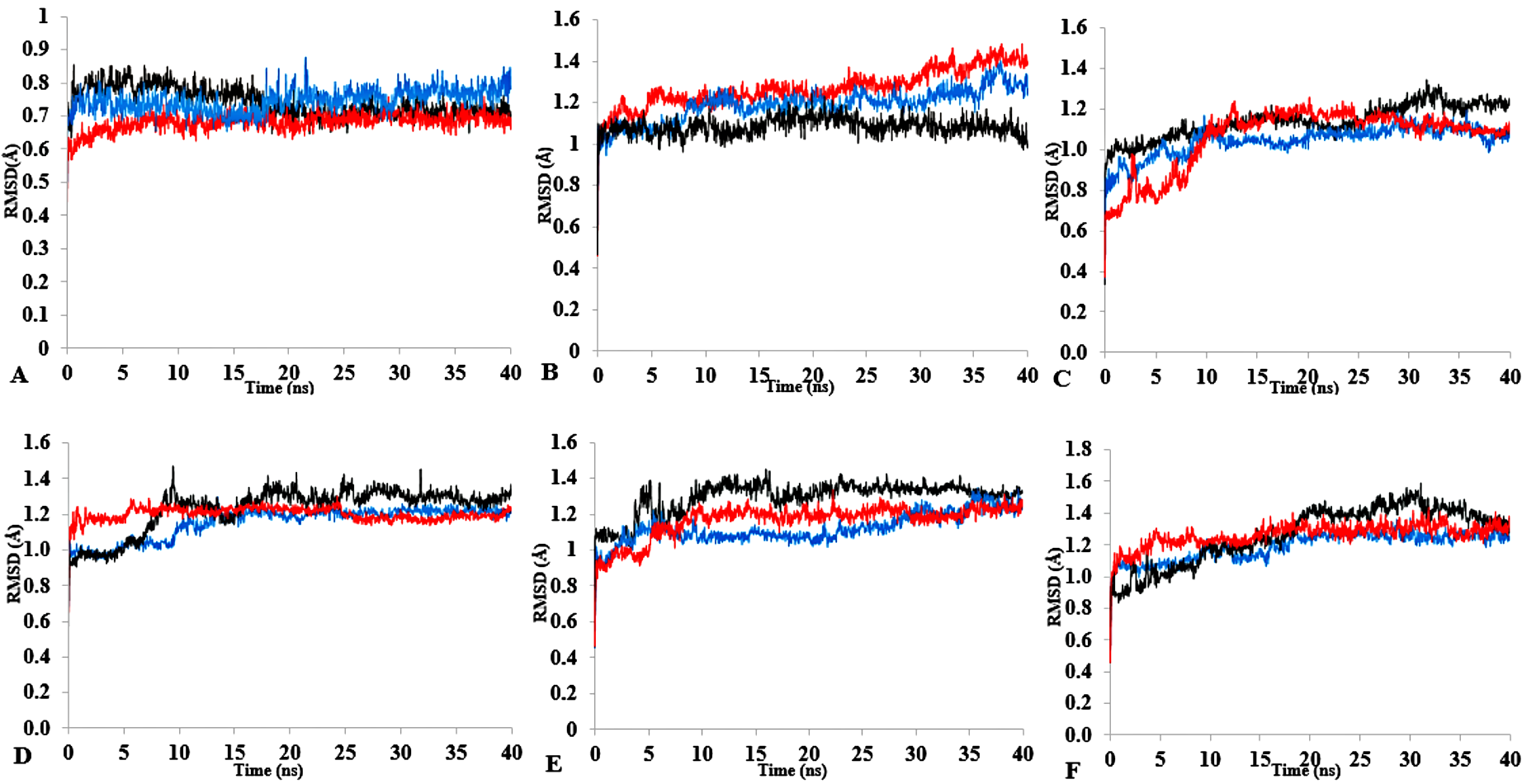

Supplement: Figure S1 — All replicate were run at different initial starting velocities determined based on the RandomSeed whereby the atom velocities are assigned randomly, using the built-in random number generator. Plots show first replicate (black), second (blue) and third (red) in (A) H2O (B) MeOH-H2O (C) EtOH-H2O (D) PrOH-H2O (E) BtOH-H2O (F) PtOH-H2O. [file peerj-05-3341-s001.png]

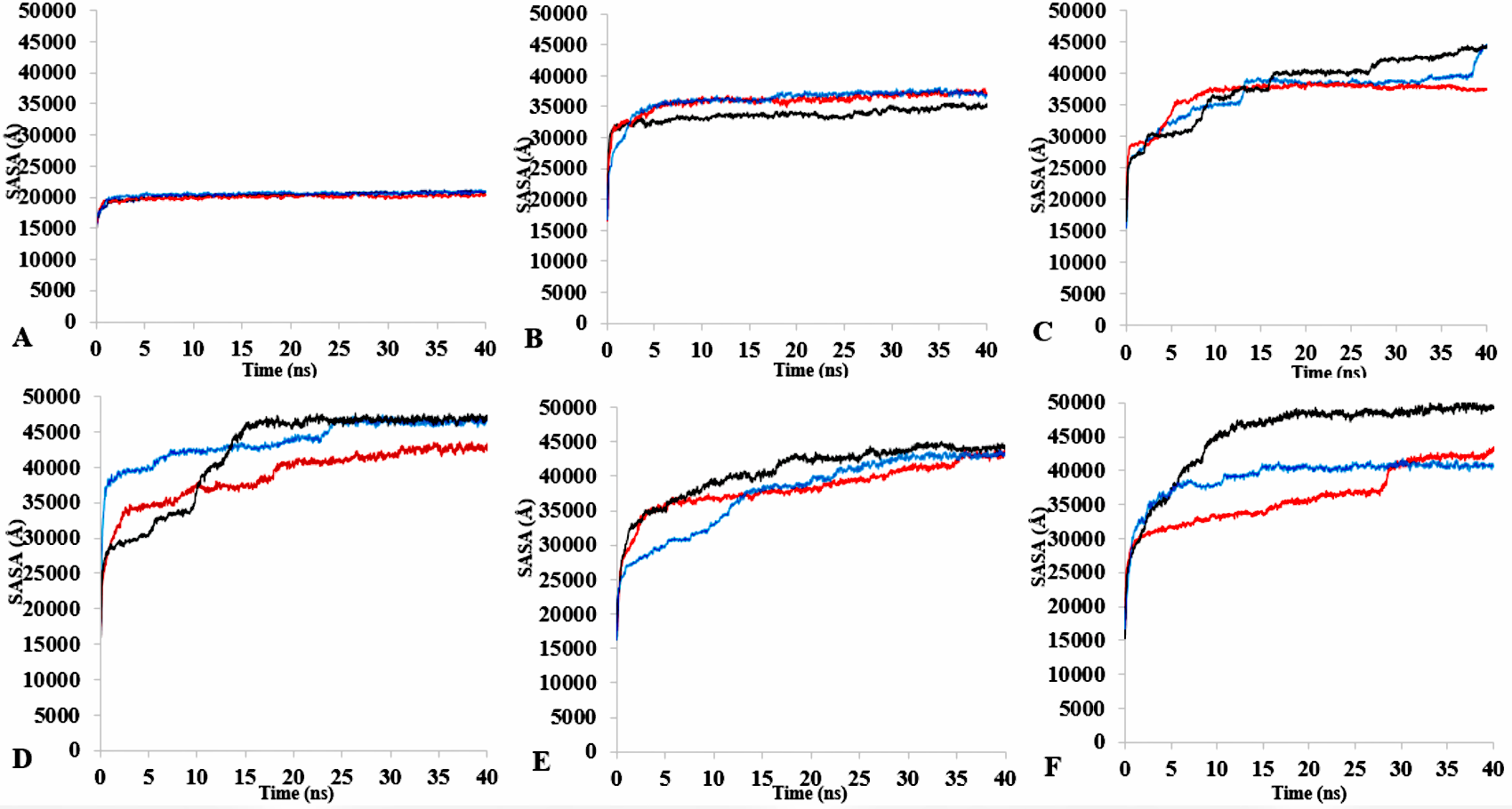

Supplement: Figure S2 — Replicates were generated for (A) H2O (B) MeOH- H2O (C) EtOH-H2O (D) PrOH-H2O (E) BtOH-H2O (F) PtOH-H2O. Plots show first replicate (red), second (black) and third (blue). [file peerj-05-3341-s002.png]

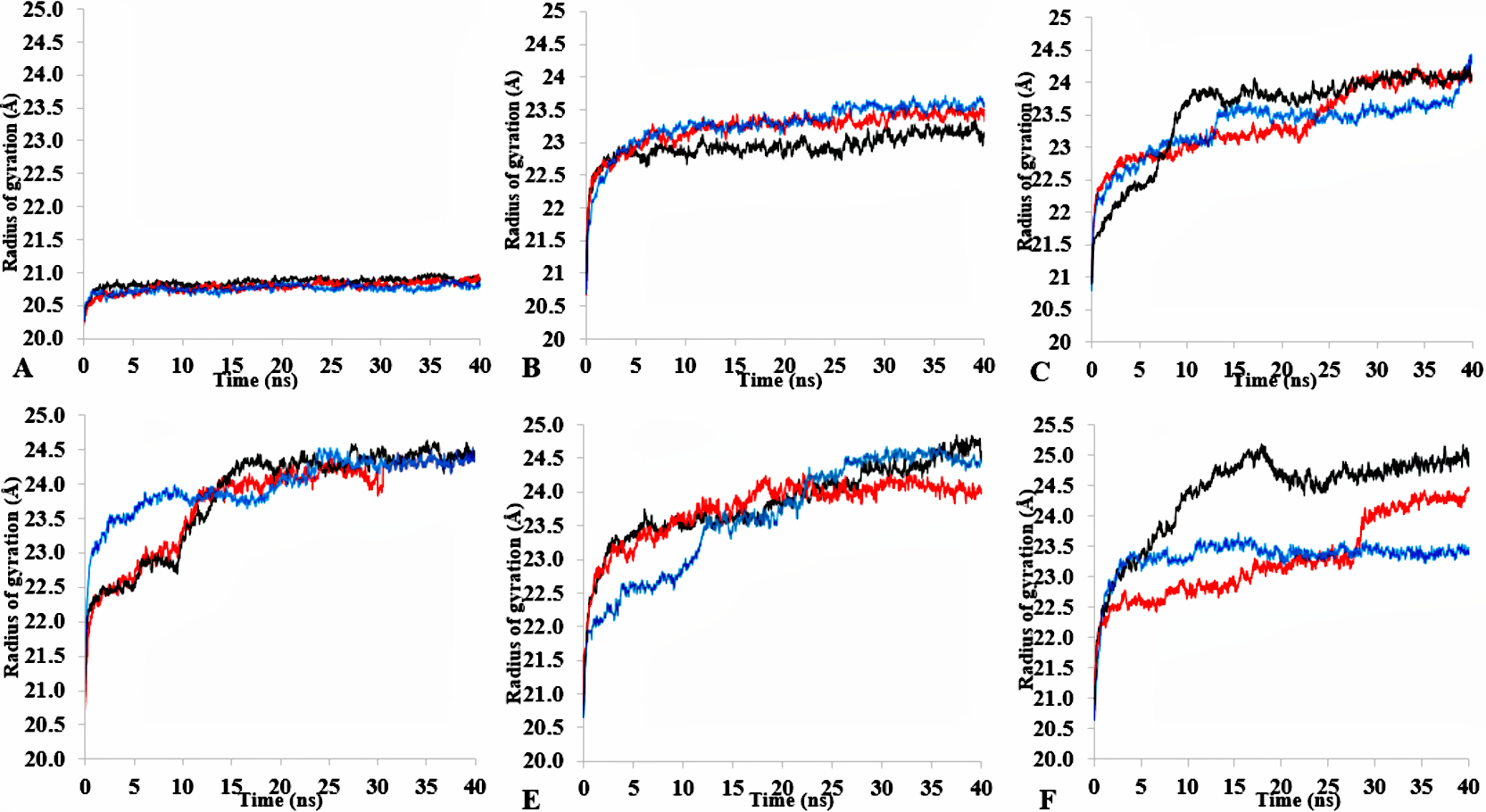

Supplement: Figure S3 — Radius of gyration which shows the same level of compactness among solvents compared to H2O. Both properties are calculated from mean average of three replicates of 40 ns simulations for (A) H2O (B) MeOH-H2O (C) EtOH-H2O (D) PrOH-H2O (E) BtOH-H2O (F) PtOH-H2O. Plots show first replicate (red), second (black) and third (blue). [file peerj-05-3341-s003.png]

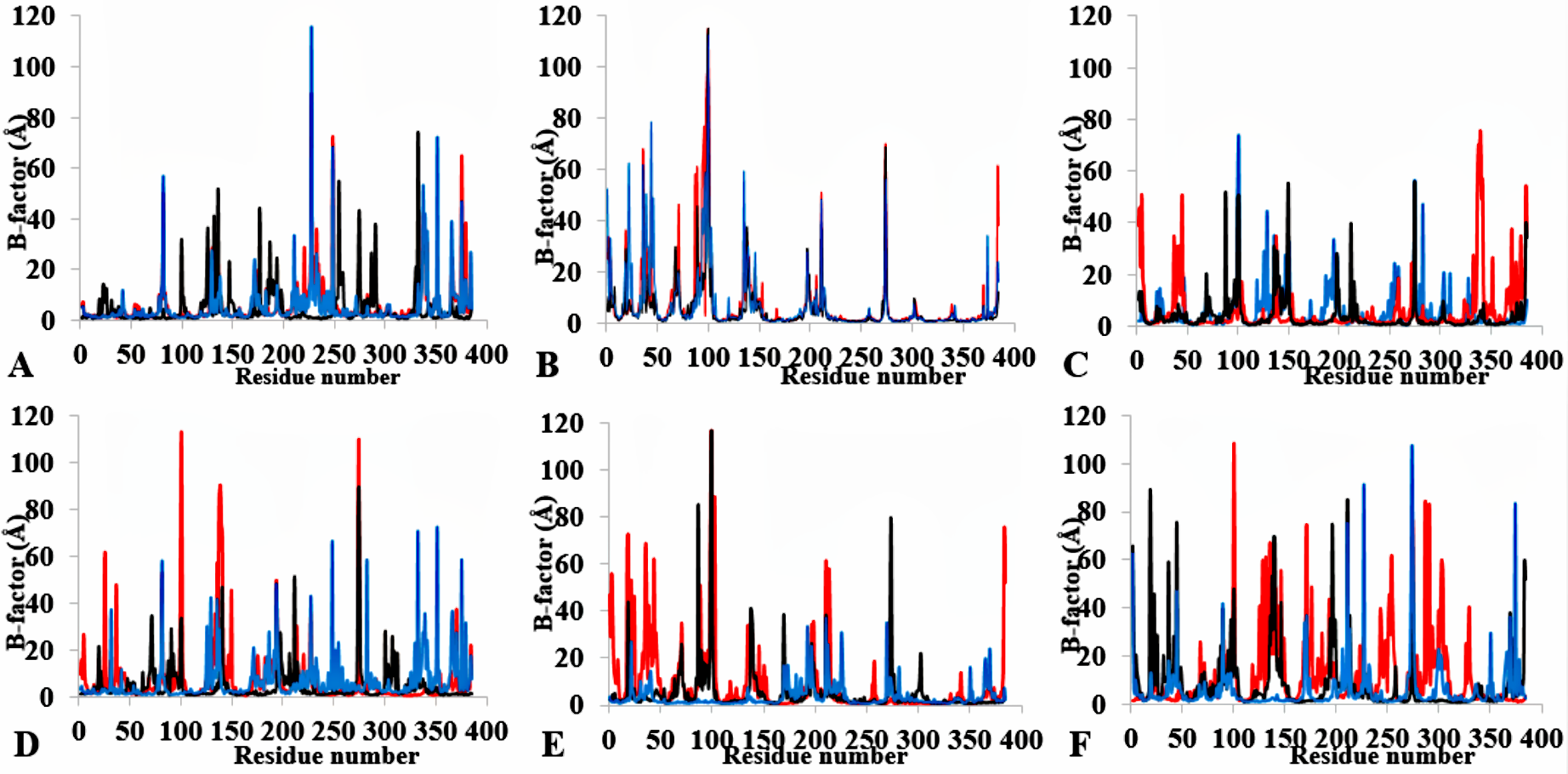

Supplement: Figure S4 — B factor of T1 lipase as a function of simulation conditions in three replicates for (A) H2O (B) MeOH-H2O (C) EtOH-H2O (D) PrOH-H2O (E) BtOH-H2O (F) PtOH-H2O. Plots show first replicate (red), second (black) and third (blue). [file peerj-05-3341-s004.png]

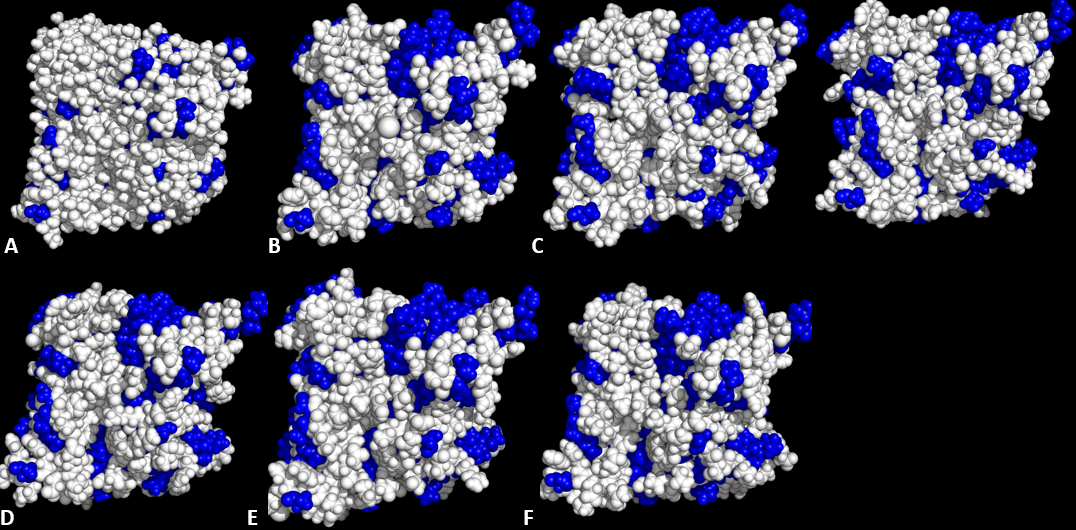

Supplement: Figure S5 — Exposed hydrophobic residues (blue) of T1 lipase of the last snapshots of 40 ns simulations as compared to (A) crystal structure (B) H2O (C) MtOH-H2O (D) EtOH-H2O (E) PrOH-H2O (F) BtOH-H2O (G) PtOH-H2O solvent mixtures. Hydrophobic residues were mapped using Open-Source PyMOL 1.7.2.1 (DeLano, 2002). [file peerj-05-3341-s005.png]

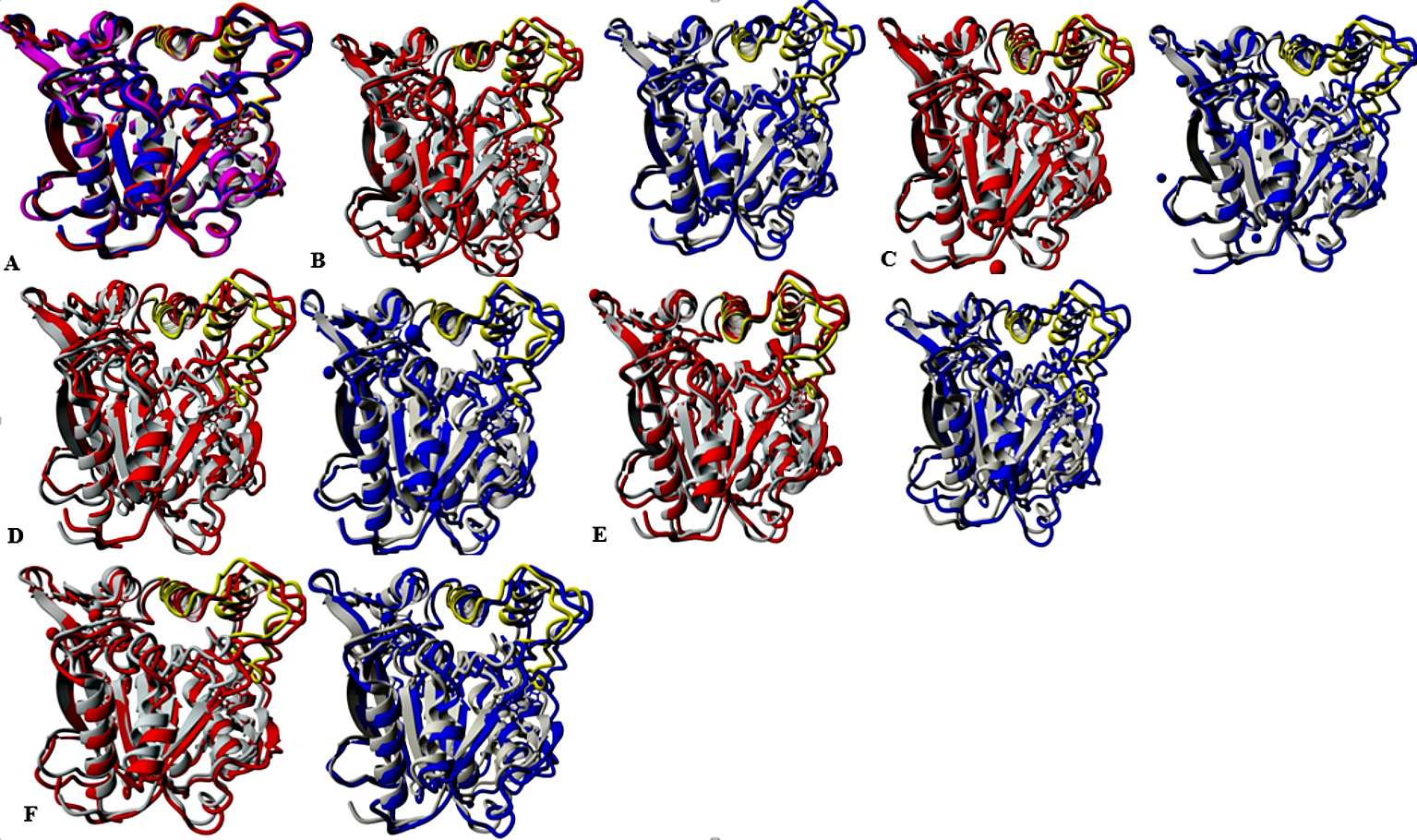

Supplement: Figure S6 — Localized structural differences are observed near the lid domain (yellow), at the beginning of the helix Asp175 all through the loop to Arg230 which shows a gradual lid opening. The first replicate is presented in Fig. 7 and other replicates are colored in red and blue for respective solvents. Water (A) replicates are superposed together with no structural differences. [file peerj-05-3341-s006.png]

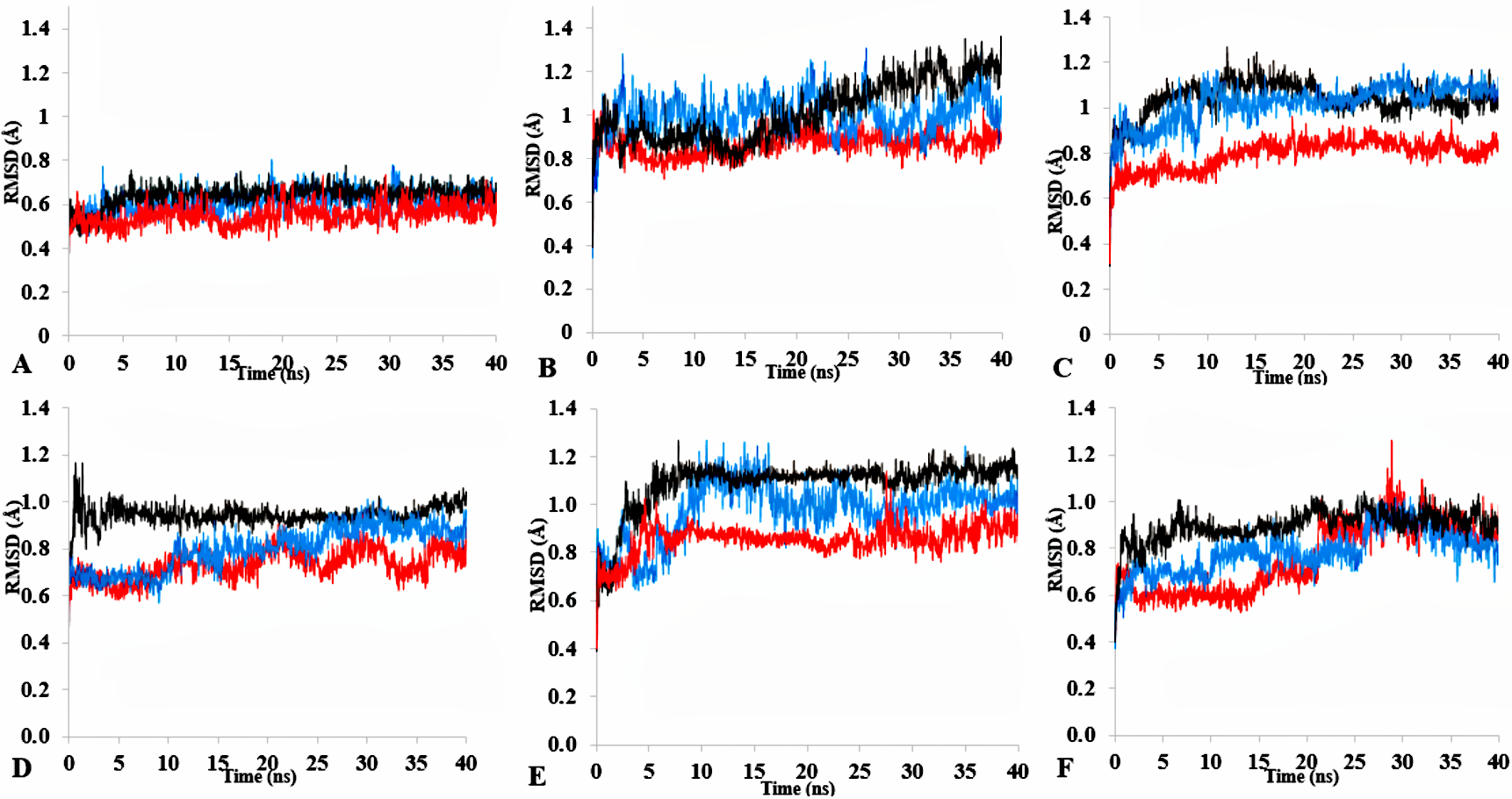

Supplement: Figure S7 — Lid domain rmsd calculated from the C α atoms of residue Asp175-Arg230 in all solvents. Solvents mixtures are H2O (A), MtOH-H2O (B), EtOH-H2O (C), PrOH- H2O (D), BtOH- H2O (E), PtOH- H2O (F). Plots show first replicate (red), second (black) and third (blue). [file peerj-05-3341-s007.png]

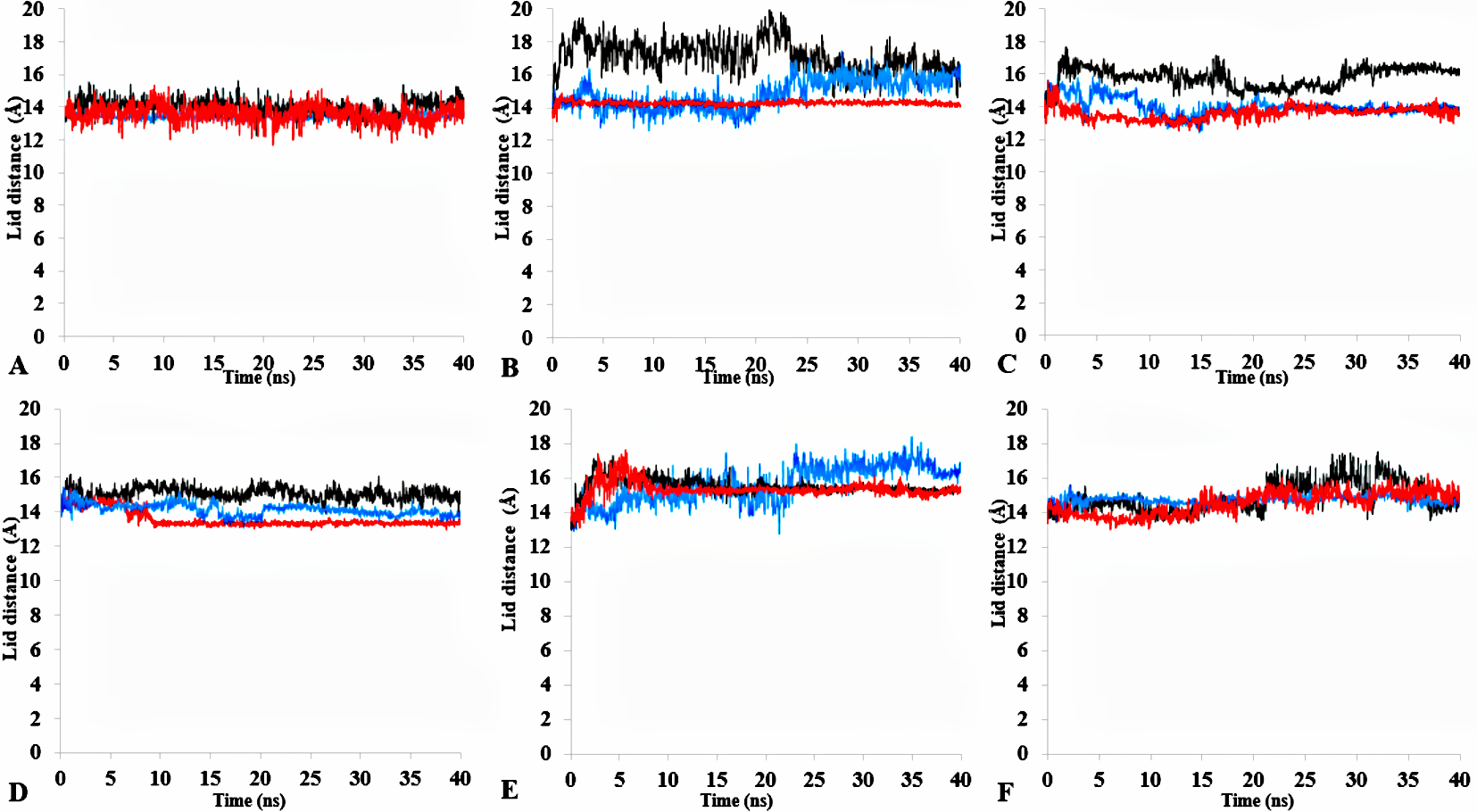

Supplement: Figure S8 — Distance of the lid measured between C α Res Asp175 and C α Res Arg230 with appreciable increase in distance of lid among all solvents. Solvents mixtures are H2O (A), MtOH- H2O (B), EtOH- H2O (C), PrOH- H2O (D), BtOH- H2O (E), PtOH- H2O (F). Plots show first replicate (red), second (black) and third (blue). [file peerj-05-3341-s008.png]

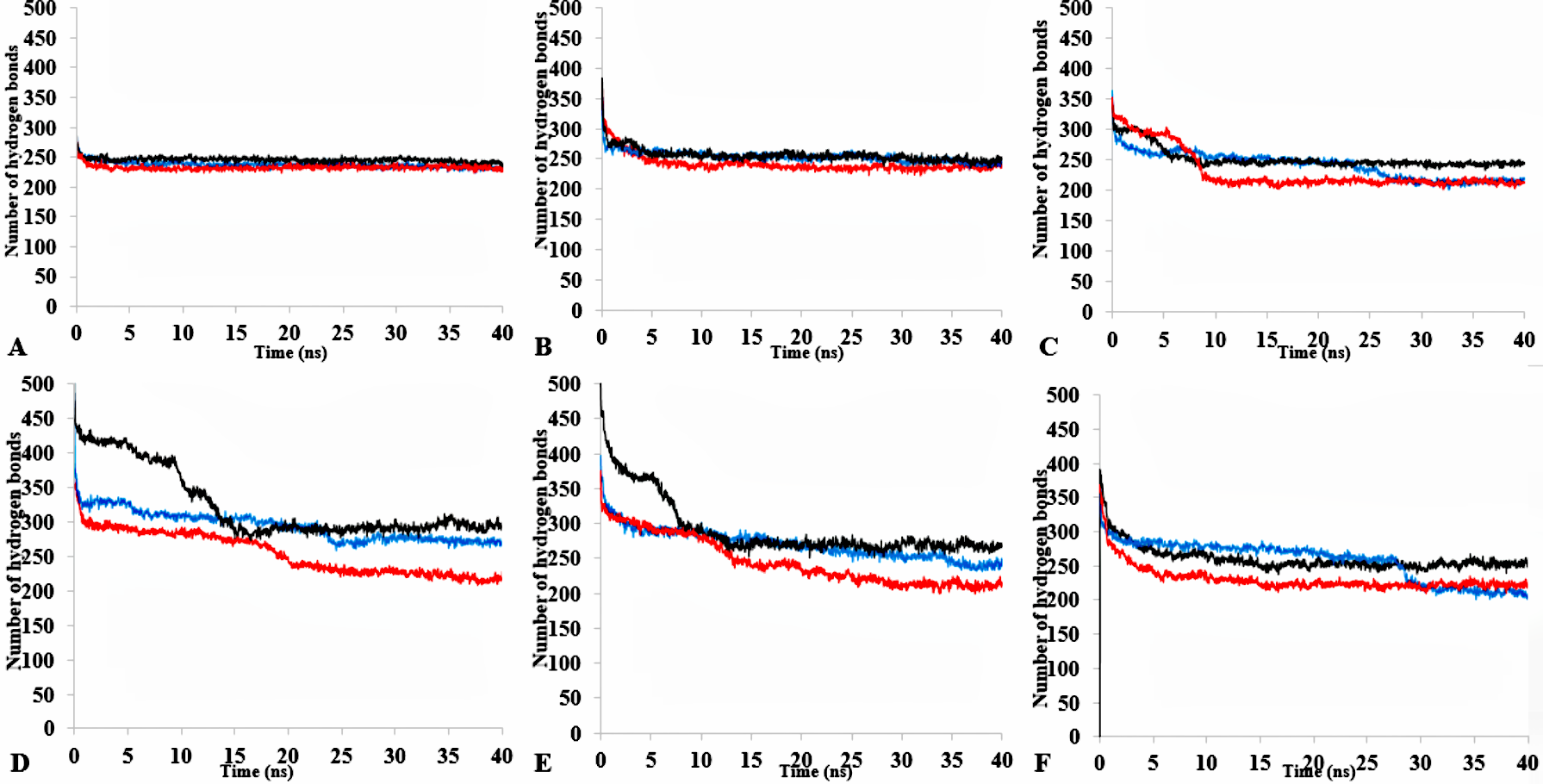

Supplement: Figure S9 — The number of hydrogen bonds within the solute as a function of the H-bond-acceptor distance in Åof 40 ns simulations. Solvent mixtures are H2O (A), MtOH-H2O (B), EtOH-H2O (C), PrOH-H2O (D), BtOH-H2O (E), PtOH-H2O (F). Plots show first replicate (red), second (black) and third (blue). [file peerj-05-3341-s009.png]

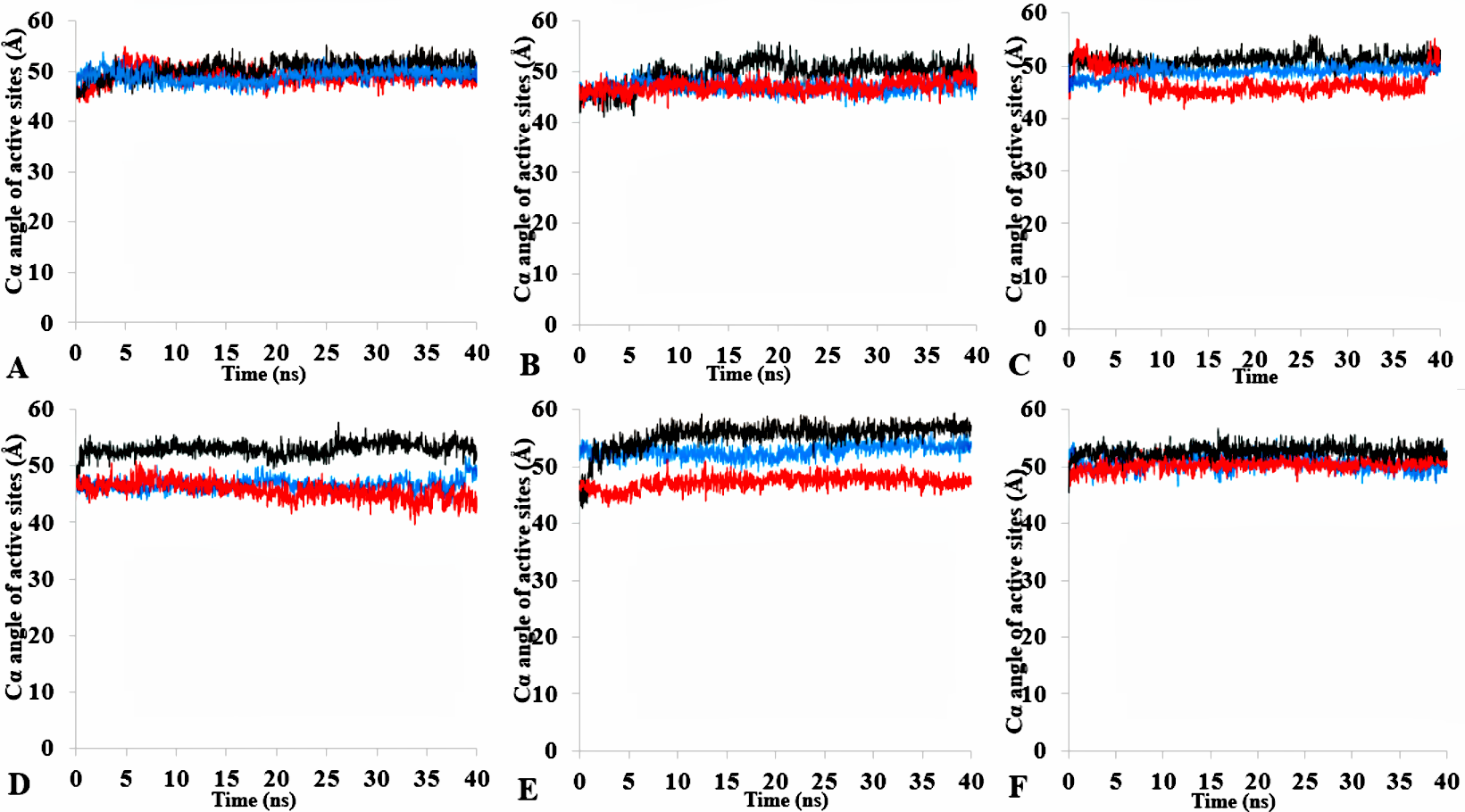

Supplement: Figure S10 — The angle between the active site residue C α atoms of Ser113, Asp 317 and His358 of H2O (A), MtOH-H2O (B), EtOH-H2O (C), PrOH-H2O (D), BtOH-H2O (E), and PtOH- H2O (F). Plots show first replicate (red), second (black) and third (blue). [file peerj-05-3341-s010.png]

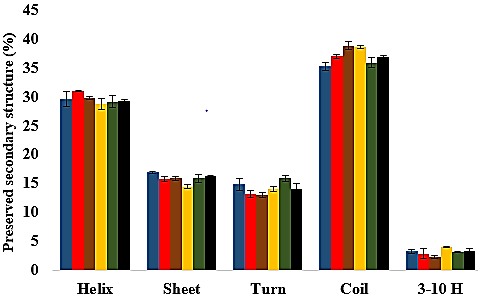

Supplement: Figure S11 — The secondary structural analysis of T1 lipase over 40 ns simulations in H2O (A), MtOH- (B), EtOH- (C), PrOH- (D), BtOH- (E) and PtOH-H2O (F) solvent mixtures. All structural elements are shown in blue (helix), red (sheet), black (turn), yellow (coil), green (3–10 helix). [file peerj-05-3341-s011.png]

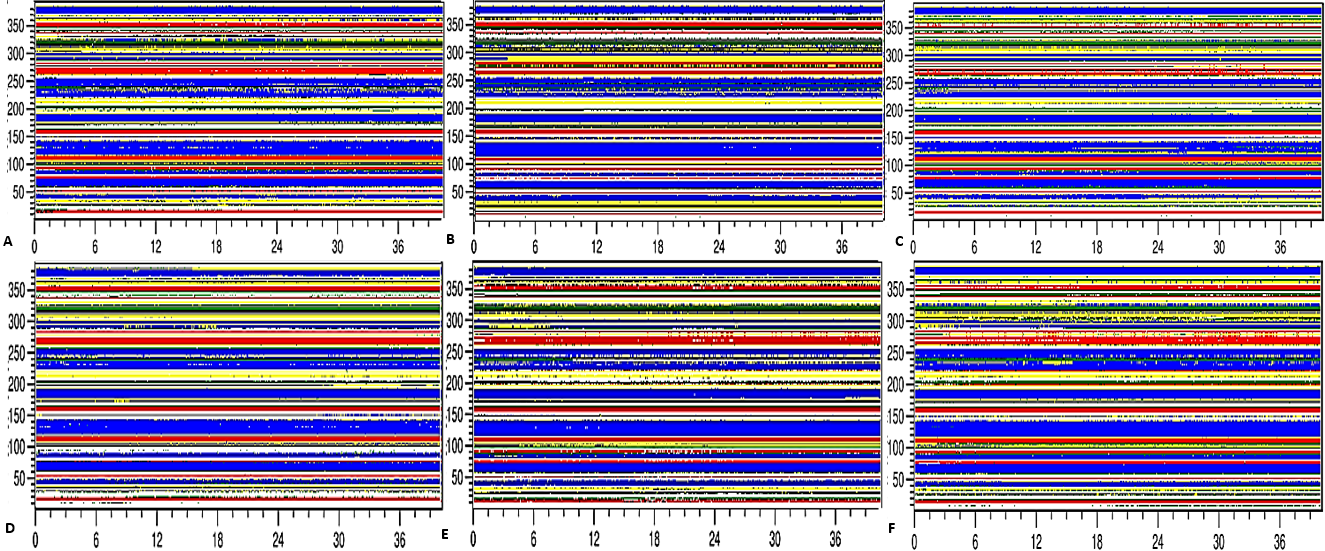

Supplement: Figure S12 [file peerj-05-3341-s012.png]
